# Research progress in inducing immunogenic cell death of tumor cells

**DOI:** 10.3389/fimmu.2022.1017400

**Published:** 2022-11-17

**Authors:** Deqian Xie, Qifei Wang, Guangzhen Wu

**Affiliations:** Department of Urology, The First Affiliated Hospital of Dalian Medical University, Dalian, Liaoning, China

**Keywords:** tumors, immunogenic cell death (ICD), ICD inducers, tumor immunotherapy, damage-associated molecular patterns (DAMPs), tumor microenvironment (TME)

## Abstract

Immunogenic cell death (ICD) is a regulated cell death (RCD) pathway. In response to physical and chemical signals, tumor cells activate specific signaling pathways that stimulate stress responses in the endoplasmic reticulum (ER) and expose damage-associated molecular patterns (DAMPs), which promote antitumor immune responses. As a result, the tumor microenvironment is altered, and many tumor cells are killed. The ICD response in tumor cells requires inducers. These inducers can be from different sources and contribute to the development of the ICD either indirectly or directly. The combination of ICD inducers with other tumor treatments further enhances the immune response in tumor cells, and more tumor cells are killed; however, it also produces side effects of varying severity. New induction methods based on nanotechnology improve the antitumor ability and significantly reduces side effects because they can target tumor cells precisely. In this review, we introduce the characteristics and mechanisms of ICD responses in tumor cells and the DAMPs associated with ICD responses, summarize the current methods of inducing ICD response in tumor cells in five distinct categories: chemical sources, physical sources, pathogenic sources, combination therapies, and innovative therapies. At the same time, we introduce the limitations of current ICD inducers and make a summary of the use of ICD responses in clinical trials. Finally, we provide an outlook on the future of ICD inducer development and provide some constructive suggestions.

## 1 Introduction

Cancer is one of the leading causes of mortality worldwide, posing a serious threat to human life and health and a severe burden on the global economy (1). According to the GLOBOCAN 2020 online database, there were 19.29 million new cancer cases and 9.96 million deaths worldwide. China had 4.57 million new cancer cases, accounting for 23.7% of the world, and 3 million deaths, accounting for 30% of the total number of cancer deaths, ranking it first in the world (2). This is because China is one of the most populous countries in the world. Unbalanced social development, regional variation in medical care, an aging population, and unhealthy lifestyle are also important reasons for the high incidence and mortality rate of tumors in recent years (3). Currently, tumor treatment is mostly based on conventional methods, such as surgical resection, chemotherapy, and radiation therapy, to inhibit or eliminate tumor cells through a wide range of tumor killing mechanisms to improve the overall survival rate of patients. However, these conventional treatment methods have significant side effects such as nausea, vomiting, hair loss, and neurotoxicity, owing to their extensive cytotoxicity and cellular resistance. The tumor microenvironment (TME) is immunosuppressed in patients with metastasis and recurrence. The efficacy of these conventional treatments is poor; tumors continue to progress, and patients have high mortality rates (4, 5).

The emergence of cancer immunotherapy has dramatically reduced the limitations of conventional treatment. Tumor immunotherapy reverses the immunosuppressive state of the TME due to factors such as reduced immunogenicity of tumor cells and the generation of immunosuppressive factors. Moreover, it restores the normal antitumor immune response, achieves the goals of controlling tumor growth, removes local or distant metastatic tumor cells, and causes long-term immune memory resistance to cancer recurrence (6). Immunotherapy for tumors includes monoclonal antibody-based immune checkpoint inhibitors, therapeutic antibodies, cancer vaccines, cell therapies, and small-molecule inhibitors. These immunotherapeutic categories have demonstrated powerful antitumor potential in clinical trials, and their high efficacy and innovative treatment modalities have created new options in the clinical treatment of tumors. They are considered to be the most promising tumor treatment methods (7).

Control of tumor progression depends on the death of tumor cells, which is usually classified as accidental cell death (ACD) and regulated cell death (RCD). *Accidental cell death* is uncontrolled cell death triggered by an injurious stimulus that exceeds the regulatory capacity of the cell, leading to the onset of cell death. RCD differs from ACD in that it has a precise molecular mechanism controlled by specific signal transduction pathways and can be regulated through genetic signals or pharmacological interventions (8, 9). RCD includes apoptosis, necrosis, and autophagy. Targeting RCD is a highly effective antitumor pathway, and different signaling pathways can be targeted through drugs that promote cell death, avoid tumor cell drug resistance, and improve antitumor efficacy (10).

**Figure 1:**
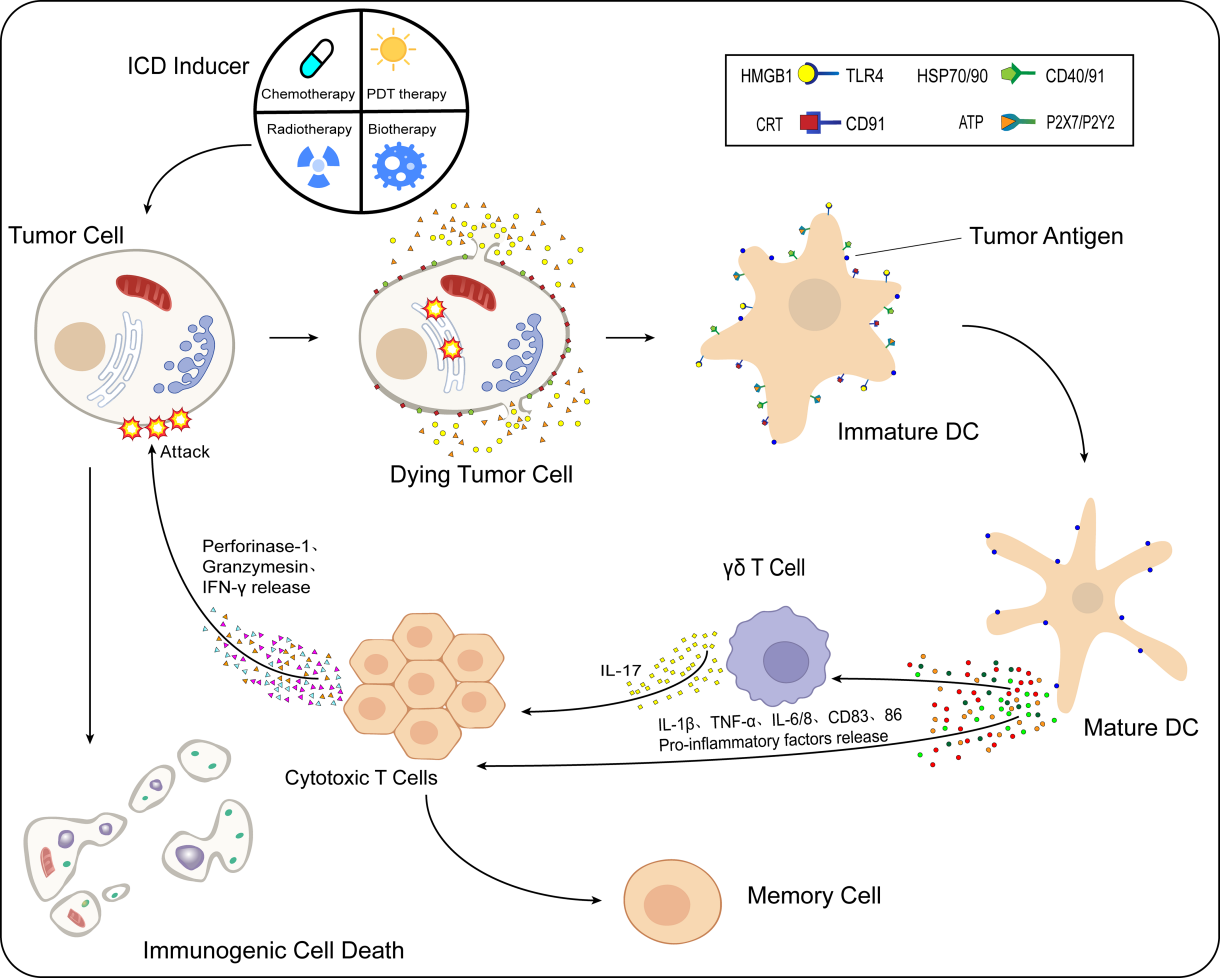
Process of immunogenic cell death (ICD). After the action of ICD inducers on tumor cells, different classes of inducers cause the onset of the endoplasmic reticulum stress response by direct or indirect means. Under the influence of multiple intracellular mechanisms of action, CRT and HSP70/90 present in the cell are exposed to the cell surface, while ATP and HMGB1 are released to the extracellular compartment *via* vesicular transport. These released DAMPs bind to immature DCs in the TME. HMGB1 binds to TLR4 receptors, CRT binds specifically to CD91, HSP70/90 binds to CD40/91 receptors, and ATP binds specifically to different P2X7/P2Y2 receptors at different concentrations. Moreover, DCs mature in the TME, while the antigen-presenting function is further enhanced. Under the action of mature DCs, levels of proinflammatory cytokines such as IL-1β, TNF-α, IL-6/8, and CD83/86 in the TME are increased. IL-1β acts on γδ T cells and CTLs, while γδ T cells release IL-17, and under the combined effect of IL-1β/17 and other cytokines, the CTLs release perforin-1, IFN-γ, granulocyte enzymes, and other substances that cause kill effects on tumor cells, and ICD occurs in tumor cells.

Immunogenic cell death (ICD) is a type of RCD and is one of the crucial mechanisms of action of tumor immunotherapy. Induced by various chemical, physical, and pathogenic sources, tumor cells generate large amounts of reactive oxygen species (ROS) to indirectly stimulate endoplasmic reticulum stress by activating specific signaling pathways or by directly acting on the endoplasmic reticulum of tumor cells to alter its structure and thus trigger stress. Driven by the endoplasmic reticulum stress response, many damage-associated molecular patterns (DAMPs) associated with ICD development are secreted or transported to the extracellular space, expressed on the cell surface, and attracted to dendritic cells (DCs) and macrophages through various pattern recognition receptors (PRRs). This promotes the maturation and activation of such cells and stimulates the antitumor immune response. The response occurs when cytotoxic T lymphocytes that are cytotoxic to tumor cells proliferate, killing the tumor cells under the action of perforin-1, interferon-γ (IFN-γ), and granulocyte enzymes (**Figure 1**). The tumor cell ICD response improves the effectiveness of cancer treatment and patient prognosis (11–13).

This review summarizes the characteristics of the ICD response in tumor cells and describes its mechanisms in detail. We also list typical DAMPs related to ICD development and some recently discovered DAMPs. We highlight the structures and their changes and effects during ICD occurrence. In addition, we summarize the recent progress in the induction of ICD responses in tumor cells over the last five years. These induction methods are categorized as: chemical sources, physical sources, pathogenic sources, combination therapies, and innovative therapies. We provide a detailed explanation of the structure, induction mechanism, and impact of the various ICD induction methods. By analyzing the mechanisms of action of these inducers, we divided them into type I and type II inducers. In addition, we outline the differences in the induction mechanisms of these two types of inducers. Of course, there are still limitations in applying ICD inducers in practice, and we make a summary of the use of ICD responses in clinical trials and identify potential problems. At the same time, we also provide an outlook on the future of ICD inducer development and provide some constructive suggestions.

## 2 Immunogenic cell death

Immunogenic cell death (ICD) is the transformation of tumor cells from non-immunogenic cells to immunogenic cells. Apoptosis occurs and stimulates an antitumor immune response *in vivo* to kill more tumor cells and hinder tumor progression (14). Immunogenic cell death was first proposed in a 2005 study that used adriamycin in a mouse tumor model *in vitro* and *in vivo*. Researchers found that the drug induced an ICD response in the mouse model, leading to DC maturation and activation, proliferation of cytotoxic T lymphocytes, which killed a large number of tumor cells (15). Immunogenic cell death responses are influenced by several factors (16). First, during tumorigenesis, tumor cells express inhibitory receptors, immunosuppressive cells release inhibitory cytokines, the TME is immunosuppressed, and the ICD response needs to overcome the immunosuppressive TME and recruit activated antigen-presenting cells (17). The second factor is the antigenicity of the responses. Infected and malignant cells can express antigenic epitopes that are not covered by thymic tolerance and are highly immunogenic, in contrast to normal cells, where this type of antigenic epitope does not exist, limiting the ability of normal cells to drive ICD response. Finally, the onset of ICD is adjuvant and is usually accompanied by the release or exposure of several DAMPs. These include calreticulin (CRT), high mobility group box 1 protein (HMGB1), adenosine triphosphate (ATP), and heat shock proteins (HSP) (16).

However, different ICD inducers do not activate the same stress response, and the DAMPs activated during the stress process differ significantly (16). During the occurrence of ICD in tumor cells, DAMPs have a significant immune function after exposure to the surface or secretion. They can interact with different pattern recognition receptors to promote various actions, such as maturation/activation of immune cells; antigen recruitment, processing and presentation; and cytokine production, ultimately contributing to the activation of anticancer immunity (18–20). However, because these DAMPs belong to different categories and have different structures, the mechanisms by which they exert their immune effects are also different. Therefore, understanding the structure of these DAMPs and the mechanisms by which they function is crucial for an in-depth study of ICD.

### 2.1 Calreticulin

Calreticulin (CRT), the major calcium-binding protein in the endoplasmic reticulum, is highly conserved, present in all cells except erythrocytes, and has biological functions such as molecular chaperone activity, regulation of Ca^2+^ homeostasis, and regulation of gene expression (21, 22). During the ICD response in tumor cells, eukaryotic initiation factor 2α (eIF2α) is phosphorylated under the influence of specific drugs or external factors, accompanied by the suspension of protein translation, which is a severe stress response of the cellular endoplasmic reticulum, with activation of pro-apoptotic caspase-3 and caspase-8, hydrolysis of endoplasmic reticulum proteins. Simultaneously, BAX and BAK, members of the pro-apoptotic Bcl-2 family, accumulate in the outer mitochondrial membrane, mediated by synaptosomal-associated protein 25 (SNAP25) and CRT is cis-transported from the endoplasmic reticulum to the Golgi apparatus and expressed on the cell surface through CRT-containing cytosolic vesicles (23, 24). The expression of CRT on the cell surface presents an “eat-me” signal and is recognized by CD91 and phagocytosed, which promotes DC maturation and activation, leading to the cross-presentation of tumor antigens and tumor-specific cytotoxic T lymphocyte responses, as well as a large-scale release of proinflammatory cytokines such as TNF-α and IL-6. The antitumor immune response is continuously enhanced and is mediated by multiple mechanisms (25–27). This suggests that translocation and exposure of CRT during ICD can trigger a robust antitumor immune response, and is an important marker of ICD-stimulated innate and adaptive anticancer immunity.

### 2.2 High mobility group box 1 protein

High mobility group box 1 protein (HMGB1) is a highly conserved nuclear protein widely distributed in mammalian cells (28). It is secreted by activated macrophages and tumor necrosis cells, is usually bound to intracellular chromatin, and is released extracellularly when cells are mechanically damaged and necrotic (29). During the ICD response in tumor cells, HMGB1 is released extracellularly to bind to pattern recognition receptors (PRRs), including Toll-like receptors and receptors of advanced glycation end products (RAGE) (30). In combination with the Toll-like receptor TLR4, HMGB1 is a proinflammatory stimulator that activates the release of proinflammatory cytokines from monocytes or macrophages and enhances antigen presentation by DCs (31). HMGB1 can increase the levels of proinflammatory cytokines such as TNF, IL-1, and IL-8. Moreover, immune cells are recruited to exert a powerful antitumor immune effect by interacting with RAGE (32). The combination of HMGB1 with Toll-like receptors and RAGE leads to the activation of nuclear factor-κB (NF-κB) (33) and concurrently increases angiogenic factor production, tumor tissue destruction, and further promotes the inflammatory response (34). Through the positive feedback mechanism of HMGB1, proinflammatory cytokines and angiogenic factors are continuously produced (31). This suggests that the extracellular release of HMGB1 is an important marker for the development of ICD response in tumor cells.

### 2.3 Adenosine triphosphate

Adenosine triphosphate (ATP) is one of the most abundant intracellular metabolites and the most crucial component in the formation of the TME, in which a large amount of ATP is released by autophagy of the tumor cells (35). In the process of ICD in tumor cells, autophagy of tumor cells can degrade damaged organelles, cytoplasmic proteins, and other materials (36). ATP is released outside the cell through the active cytosol of ATP-containing vesicles. In the TME, ATP acts on P2 purinergic receptors expressed on the surface of the tumor and host, and different ATP levels and types of P2 purinergic receptors have different effects (37, 38). The ATP concentration required to activate P2X7 is greater than 100 μmol/L (39), and the P2X7 receptor can activate cytotoxic T lymphocytes. When the ATP concentration is less than 1 μmol/L (39), ATP can activate the P2Y2 receptor, and ATP acting on the P2Y2 receptor can send a “find me” signal to DCs and macrophages, promoting DC activation and maturation and the expansion of macrophages (40). Concurrently, lower concentrations of ATP can exert anti-inflammatory effects, mainly through activation of the NLRP3 inflammasome and massive release of proinflammatory cytokines, such as IL-1β and IL-18, mediating immunostimulatory effects (41, 42). The extracellular release of ATP enhances the host antitumor immune response, and many tumor cells are killed by multiple mechanisms, suggesting that the extracellular release of ATP is an important marker of ICD response in tumor cells.

### 2.4 Heat shock proteins

Heat shock protein (HSP) is a highly conserved protective protein that can be synthesized in large quantities under specific circumstances. It helps cells maintain normal physiological activities by refolding damaged proteins or degrading damaged proteins by acting on the proteasome, and has some anti-apoptotic ability (43, 44). The heat shock proteins involved in tumor cell ICD are mainly HSP70 and HSP90 (45). HSP70 is one of the most crucial heat shock proteins involved in protein folding and transport. Its function lies in its ability to play a protective role against various cellular stresses and regulate intracellular apoptotic signaling. In terms of immune function, HSP70 promotes the release of proinflammatory cytokines, increases the expression of co-stimulatory molecules, and regulates the immune function together with other immune molecules (46). HSP90 is a tumor marker involved in important physiological processes, such as invasion, metastasis, angiogenesis, and apoptosis in tumor cells, as well as in protein synthesis and degradation. HSP90 plays an important role in inhibiting apoptosis and promoting cell survival (47, 48). During the tumor cell ICD response, antigens bind to HSP70 and HSP90 to form a complex, which stimulates the uptake of tumor antigens and maturation and activation of DCs and NK cells. HSP90 binds to the LDL receptor protein CD91 to promote the cross-presentation of immune cells, and HSP70 binds to the co-stimulatory molecule CD40 to activate cytotoxic T lymphocytes (45, 49, 50). The expression of heat shock proteins further enhance host antitumor immune function during ICD, suggesting that heat shock proteins are important DAMPs for ICD.

### 2.5 Interferon

Interferons are cytokines with various effects, such as inhibition of cell division and antiviral and antitumor activities. Type I interferons, including IFN-α and IFN-β, control the growth of viruses, activate NK cells and macrophages, and promote DC maturation and activation. IFN-γ is the only member of the type II interferon family. IFN-γ can increase the activity of NK cells and macrophages and enhance the antigen presentation ability of antigen-presenting cells by upregulating the expression of MHC I/MHC II molecules. In addition, IFN-γ signaling can contribute to DC maturation and promote the massive expression of co-stimulatory molecules, including CD40/80/86 and CCR7 (51–54). During ICD, cyclic guanosine monophosphate (GMP)-adenosine monophosphate (AMP) synthase (cGAS) activates STING signaling. The cGAS-STING signaling pathway can stimulate IFN-I expression and initiate a powerful type I interferon response (30). It enhances the cytotoxicity of cytotoxic T lymphocytes and NK cells and promotes the cross-presentation of DCs (55, 56), while promoting the secretion of proinflammatory cytokines by macrophages (57). Moreover, it inhibits the function of immunosuppressive cells and improves the TME (58). The enhanced host antitumor immune response and death of many tumor cells suggest that interferon is a vital DAMP in ICD development. Determining the interferon content can indirectly determine whether tumor cells are undergoing an ICD response.

These five typical DAMPs are the current gold standard for predicting ICD response induced by antitumor therapy. In addition to these typical DAMPs, recent studies have identified membrane annexin A1(ANXA1) (30) and spliceosome-associated protein 130 (SAP130) (50). However, the mechanism of these newly discovered DAMPs and ICD development is still unclear, and the number and types of DAMPs insufficient to confirm the efficacy of antitumor drugs. Therefore, this area needs to be explored in future studies.

## 3 Conventional methods of ICD induction

**Figure 2:**
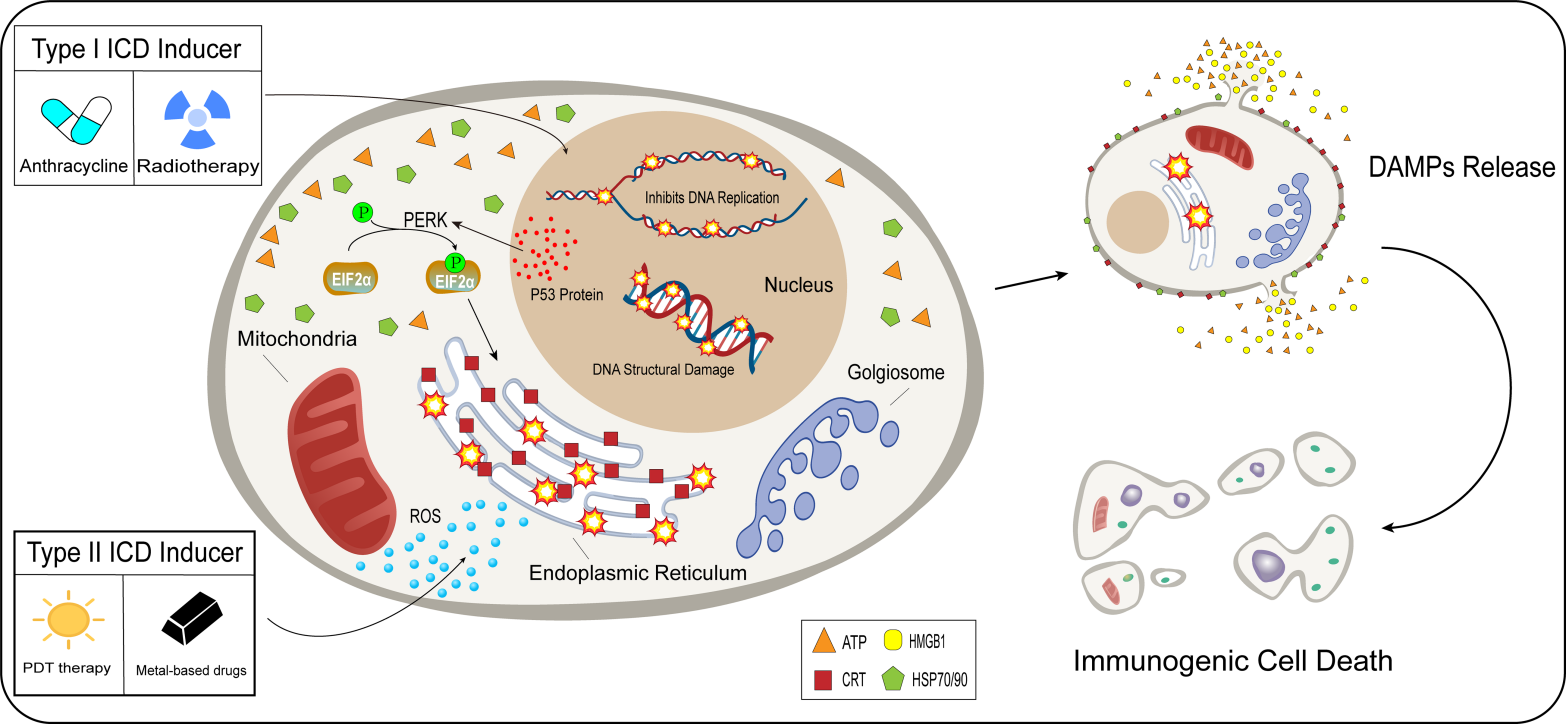
Difference between type I and type II inducers. After acting on tumor cells, type I inducers (anthracyclines, radiotherapy), affect the normal functions of tumor cells, including hindering DNA replication and promoting the breaking of the DNA structure in the nucleus. Type I inducers can increase the expression of P53 protein and enhance protein stability. p53 protein is involved in the activation process of PERK signaling in tumor cells, EIF2α undergoes phosphorylation, and the endoplasmic reticulum undergoes a stress response under the influence of a series of actions. Unlike type I inducers, type II inducers (PDT therapy, metal-based drugs), generate ROS in large quantities in the cytoplasm and selectively act on the endoplasmic reticulum, which undergoes an oxidative stress response. Type I and type II inducers lead to stress responses in the endoplasmic reticulum through indirect and direct pathways, which trigger the release of DAMPs and induce immunogenic cell death.

The development of ICD in tumor cells usually requires the induction of mediators, including chemical drug inducers and physical induction methods. They induce tumor cell death or apoptosis by affecting various stages of tumor development, promoting cytokine secretion, and improving the TME (59). Based on the mechanism and effect of induction mediators, inducers are divided into type *I* and type *II*. Most of the inducers currently used in the clinical treatment of cancer or preclinical studies are type I inducers, including anthracyclines, oxaliplatin, and radiotherapy. Type I inducers induce endoplasmic reticulum stress response secondary to tumor cell development by affecting the process of tumor cell development and inducing ICD-related immunogenicity (60–62). Type II inducers differ from type I in that they act selectively on the endoplasmic reticulum, altering its homeostasis, triggering an endoplasmic reticulum stress response, and inducing ICD. These include photodynamic therapy and oncolytic viruses (12, 63)(**Figure 2**). Compared with type I inducers, type II inducers can increase the secretion of DAMPs, have higher transport efficiency, and have superior effects compared with those of type I inducers (64).

### 3.1 Chemical sources of ICD induction

Chemical agents are the most commonly used inducers in clinical oncology treatment or basic research to induce ICD development, including classical ICD inducers such as anthracyclines (adriamycin, mitoxantrone, etc.), oxaliplatin, 5-fluorouracil, and cardiac glycosides, which have been used in clinical treatment (60, 62, 65, 66). They also include some newly discovered inducers used in basic research that can induce ICD; they have not yet been tested in clinical trials. The induction mechanisms and effects of these two types of chemical inducers are summarized in **Table 1**.

**Table 1:** Chemical sources of ICD induction.

Inducer classification	Inducer	Cell lines	Induction mechanism	Induction effect	References
Type I	Platinum-based anticancer substances	LLC; KLN 205; H22; HepG2; CT26; HCT116	CRT exposure; release of DAMPs such as HMGB1, ATP.	ICD induction; altered immunogenic TME; cytotoxic T lymphocyte recruitment; DC maturation; increased CD80 and CD86; decreased number of Tregs; inhibition of TGF-β secretion	(60, 67, 68)
Type I	Anthracycline anticarcinogens	LNCaP; 22RV1; PC-3; RM-1	CRT exposure; ATP, HMGB1, and other DAMPs release; EIF2α phosphorylation; PERK/GCN2 pathway activation; P53 protein non-dependent pathway	ICD occurrence; enhanced P53 protein stability; enhanced antitumor immunity	(62, 69)
Type I	Taxanes	ID8; MDA-MB-231	Irreversible cell damage; IKK2 phosphorylation; SNAP23 phosphorylation; activation of TLR4/IKK2 signaling; CRT exposure; release of DAMPs such as ATP, HMGB1	ICD induction; CXCL10 upregulation; significantly higher expression of MHC II and CD86; DC maturation; altered TME; increased IFN-γ production	(70, 71)
Type I	Cardiac glycosides	MDA-MB-231; mcf7; t47d	CRT exposure; release of DAMPs such as ATP, HMGB1, and heat shock proteins; PERK axis activation; ER stress response.	DC maturation and activation; cytotoxic T lymphocyte recruitment; ICD induction; increased CD80 and CD86; significantly increased IL-2 and IFN-γ levels; decreased IL-10 expression.	(66)
Type I	5-Fluorouracil	HepG2; KYSE 30	CRT exposure; release of DAMPs such as ATP, HMGB1.	ICD induction; DCs maturation; increased proliferation of cytotoxic T lymphocytes; elevated HLA class I surface expression; upregulation of CD80 and CD86	(65, 72)
Type I	CDK12/13 specific inhibitors	MDA-MB-231; MCF7; T47D; 4T1	IRE1 phosphorylation; EIF2α phosphorylation; ER stress response; PERK pathway activation; CRT exposure; ATP, HMGB1, and other DAMPs release	ICD induction; DCs maturation; CD80, CD86 upregulation; infiltrating cytotoxic T lymphocyte activation; IL-1β, TNF-α, IL-6 level elevation; alteration of the TME	(73)
Type I	AXL Inhibitors	HCC827; H1975.	EGFRI resistance; CRT exposure; release of DAMPs such as ATP, and HMGB1; irreversible cellular damage; inhibition of cellular transcription	ICD induction; cytotoxic T lymphocyte recruitment; antitumor immune enhancement	(74, 75)
Type I	PLK1 inhibitor	LLC	Irreversible cell damage; blocked division cycle; CRT exposure; release of DAMPs such as ATP, HMGB1	ICD induction; DCs maturation; increased surface expression of CD80, CD86, and MHCII; cytotoxic T lymphocyte recruitment.	(76)
Type I	ADI-PEG20	MC38; MDA-MB-231	CRT exposure; release of DAMPs such as ATP, HMGB1; blocking cell cycle	ICD induction; alteration of the TME.	(77)
Type I	BET-targeting agents	HCT-116	Degradation of DR5; CRT exposure.	ICD induction; increased cytotoxic T cell infiltration; decreased immunosuppressive Tregs	(78)
Type I	Mitochondrial uncouplers	EOC	CRT exposure; release of DAMPs such as ATP, HMGB1; ER stress response.	ICD induction; increased proportion of cytotoxic T lymphocytes; proinflammatory cytokine production; antitumor immune enhancement	(79)
Type I	Belantamab mafodotin	NCI-H929; EL4	CRT exposure; release of DAMPs such as ATP, HMGB1, and heat shock proteins; eif2α phosphorylation; PERK pathway activation; ER stress response.	NK cell increase; ICD induction; activation of tumor-specific cytotoxic T cells; enrichment of tumor-infiltrating lymphocytes; DCs maturation; CD40, CD86 increase	(80)
Type II	Iridium-based anticancer substances	A549; A549R; LLC; MDA-MB-231; CT-26; HLF; BEAS-2B	CRT exposure; HMGB1, ATP and other DAMPs release; CHOP upregulation; eIF2α phosphorylation; ROS-induced ER stress-based response; activation of caspase 3/7 signaling	ICD induction; cytotoxic T lymphocyte recruitment; significant reduction in Foxp3^+^ T cells (CD3^+^CD4^+^Foxp3^+^ T lymphocytes)	(81)
Type II	Ruthenium-based anticancer substances	HCT-15; HCT-116; HT-29	PERK signaling activation; enhanced autophagy; irreversible cellular damage; CRT exposure; release of DAMPs such as HMGB1, ATP, and heat shock proteins.	ICD induction; DCs maturation; cross-presentation to cytotoxic T cells.	(82, 83)
Type II	Copper anticancer substances	CT26; 4T1; HCT116	CRT exposure; ER stress response based on ROS stimulation; ATP, HMGB1, and other DAMPs release; JNK, NF-κB, PI3K signal transduction	ICD induction; activation of cytotoxic T-cells; reduced tumor size	(84, 85)
Type II	Non-steroidal anti-inflammatory substances	HCT-116; H29	CRT exposure; NSAID-induced ER stress; DR signaling activation	ICD induction; increased tumor-infiltrating lymphocytes (TILs); decreased immunosuppressive regulatory T cells (Tregs)	(86)
Type II	PKHB1	CCL-119CCRF-CEM; CRL-1582MOLT-4; MDA-MB-231; MCF7; 4T1	Activated caspase signaling; CRT exposure; ATP, HMGB1, and other DAMPs release; Ca2^+^-dependent cell death; ROS-stimulated ER-based stress response	ICD induction; promotion of cytotoxic T cell infiltration; DC maturation; reduction in immunosuppressive Tregs	(87, 88)

A549, human lung epithelial carcinoma cells; A549R, cisplatin-resistant human lung epithelial carcinoma cells; LLC, Lewis lung cancer; MDA-MB-231, MCF7, T47D, triple-negative human breast cancer cells; CT-26, MC38, mouse colon cancer cells; HLF, normal human lung fibroblasts; BEAS-2B, normal human lung epithelial cells; KLN 205, mouse lung squamous carcinoma cells; H22, mouse hepatocellular carcinoma cells; HepG2, human hepatocellular carcinoma cells; HCT-15, RKO, HCT-116, HT-29, human colon cancer cells; 4T1, mouse breast cancer cells; SKOV3, A2780, human ovarian cancer cells; KYSE 30, human esophageal squamous carcinoma cells; HCC827, H1975, human non-small cell lung adenocarcinoma cells; CCL-119 CCRF-CEM, CRL-1582 MOLT-4, human acute lymphoblastic leukemia; EOC, human epithelial ovarian cancer cells; NCI-H929, human myeloma cells; EL4, mouse lymphoma cells; TME, tumor microenvironment.

#### 3.1.1 Metal-based inducers

Metal-based inducers are the most commonly used chemical agents that induce ICD in tumor cells. In addition to oxaliplatin (60), new metal-based inducers have been discovered, including iridium (81), ruthenium (82), and copper (84). These new metal-containing compounds have been shown to release DAMPs in basic research experiments, suggesting their potential to induce ICD in tumor cells. Among them, platinum-based metal compounds were the first discovered ICD metal inducers used in clinical treatment. Oxaliplatin is the most representative platinum metalloid, a third-generation platinum drug with low nephrotoxicity and neurotoxicity, and is the most potent platinum drug for killing tumor cells (89). Oxaliplatin, a type I inducer, induces cell death indirectly by DNA damage and intracytoplasmic effects on the endoplasmic reticulum (60). It has excellent therapeutic effects on lung (67), liver (68), colon (60), and other cancers.

In lung cancer mouse model experiments (67), a significant surface CRT exposure and a significant increase in the release of HMGB1 and ATP was found in oxaliplatin-treated cells. Exposure to these DAMPs promoted the maturation of DCs, increased the number of CD8^+^ T cells and the infiltration of tumor cells, decreased the number of regulatory T cells, improved the immunogenic TME, effectively inhibited the growth of tumor cells, and promoted tumor cell death. Tumor cell response was dose-dependent on the oxaliplatin-induced drug with a stronger response at higher concentrations. Lower the viability of tumor cells is associated with a higher the apoptosis rate and a significantly improved survival rate of hosts (68).

PT-112, a combination of platinum and pyrophosphate, is a novel platinum derivative that is capable of massive accumulation at the site of tumorigenesis. Cells treated with PT-112 showed increased CD8^+^ cytotoxic T lymphocyte infiltration and decreased Treg-dependent immunosuppression, along with the release of immunostimulatory DAMPs, suggesting that tumor cells undergo ICD (90). In clinical trials, PT-112 was shown to be significantly effective in patients with primary/metastatic tumors who failed conventional therapy, improving patient survival (91, 92).

Ruthenium, iridium, and copper metal compounds are novel ICD inducers. They are used in lung (81), colorectal (82), and breast cancer (84) cell model experiments that exhibit ICD-specific features, including surface exposure to CRT and increased release of DAMPs such as extracellular ATP and HMGB1. These three metal compound inducers entered the endoplasmic reticulum of tumor cells and induced the release of the DAMPs mentioned above by increasing the level of intracellular reactive oxygen species (ROS) and oxidative stress in the endoplasmic reticulum, leading to the development of ICD in tumor cells. Based on this mechanism, these three novel metal compounds can be classified as type II inducers (81, 82, 84).

Experiments on lung cancer cells showed that in response to iridium-like metal compound inducers, tumor cells underwent upregulation of CHOP and phosphorylation of EIF2α (81), which are typical manifestations of the endoplasmic reticulum stress response. Endoplasmic reticulum stress led to the release of Ca^2+^ from the endoplasmic reticulum, altering the amount of ER- mitochondrial Ca^2+^. Excess Ca^2+^ induced opening of the mitochondrial permeability transition pore, breaking the stability of the intracellular electron transport chain and generating excess ROS. In response to the combined endoplasmic reticulum stress and mitochondrial dysfunction, tumor cells undergo ICD, cytotoxic T lymphocytes increased, regulatory T cells decreased significantly, and tumor development was inhibited significantly.

Plecstatin-1 is a ruthenium metal compound derivative that can break the tumor cytoskeleton leading to structural alterations of the tumor. In addition, the same ruthenium metal compound can induce ROS generation and endoplasmic reticulum stress, thereby contributing to ICD development. Furthermore, DAMPs associated with ICD development, HSP70 and HSP90, enter the extracellular space and bind to antigen-presenting cell surface receptors, promoting DC activation and maturation and further enhancing tumor-associated immune effects (82, 83).

Disulfiram (DSF), a drug used to control alcoholism, acts on tumor cells in a Cu-dependent manner and can exert an inhibitory effect on tumor cell proliferation. Its pharmacological mechanisms include induction of oxidative stress through PI3K and NF-κB signaling pathways and inhibition of proteasomal activity, a dose-dependent increase in DAMPs associated with ICD development, cell activation of toxic T lymphocytes, ICD response, and further promotion of tumor cell death (85). In conclusion, these metal compound inducers can induce ICD responses in tumor cells, inhibit tumor cell development, and promote tumor cell death, providing a novel approach for patients with primary or secondary tumors when conventional treatment fails.

#### 3.1.2 Anthracycline inducers

Anthracycline anticancer drugs, including adriamycin and mitoxantrone, are among the most commonly used drugs in clinical oncology treatment. Their mechanisms of action include inhibition of DNA replication and RNA synthesis through DNA embedding, inhibition of topoisomerase II to hinder DNA replication, and generation of free radicals to break the structure of cellular DNA and proteins (93).

Mitoxantrone (MTX), a synthetic derivative of adriamycin, causes DNA cross-linking and breakage through insertion, thereby blocking DNA and RNA synthesis (94). In prostate cancer cell experiments, MTX acting on tumor cells showed typical features of DAMPs, including exposure to CRT, increased extracellular release of ATP and HMGB1, and induction of the ICD response in tumor cells. Moreover, the mechanism of action of MTX lies in the enhanced stability of P53 protein, which is also involved in activating the PERK signaling pathway. Activation of the PERK signaling pathway induces EIF2α phosphorylation in prostate cancer cells through the P53 non-dependent pathway, suggesting the occurrence of the endoplasmic reticulum stress response, which is an important trigger for the ICD response in tumor cells. Additionally, MTX enhanced the phagocytic ability of DCs and strengthened anti-prostate tumor immunity (62). One study showed that MTX combined with proteasome inhibitors resulted in enhanced prostate tumor growth and decreased overall survival in experimental mice, suggesting that the MTX-induced ICD process requires proteasome activation and that proteasome inhibitor utilization significantly reduces the release of ICD-associated DAMPs (69).

#### 3.1.3 Taxol inducers

The most common chemical agent among the Taxol inducers is paclitaxel, which is currently the first-line regimen used in the clinical treatment of breast cancer. It is capable of stabilizing microtubules, causing mitotic cell death, and blocking the G2-M phase cell cycle (95). In experiments with ovarian cancer cells (70), paclitaxel-induced CRT exposure increased ATP expression, extracellular HMGB1 and ANXA1 expression, and other DAMPs in mouse ovarian cancer cells. Paclitaxel can also activate the PERK signaling pathway and induce the phosphorylation of EIF2α, which can induce the secretion of extracellular ATP in tumor cells. In conclusion, the induction of ICD response in ovarian cancer by paclitaxel was associated with TLR4 non-dependent and TLR4-dependent pathways, which provides a theoretical basis for the clinical application of paclitaxel in ovarian cancer.

#### 3.1.4 Cardiac glycoside inducers

Cardiac glycosides are metabolic substances isolated from plants or animals that regulate the rate of cardiac contraction by acting on the cellular sodium-potassium ATPase pump, and are therefore widely utilized in the treatment of various cardiac diseases (96).

Recent studies have demonstrated the anticancer activity of cardiac glycosides. The cytotoxic effects of cardiac glycosides on tumor cells include cell-specific and dose-dependent inhibition of tumor cell growth and induction of apoptosis, inhibition of the MAPK/Wnt/PAM signaling pathway, inhibition of the G2/M cell cycle in tumor cells, induction of DNA damage, and inhibition of the PI3K/AKT/mTOR signaling pathway to promote apoptosis (97). Cardiac glycosides have been found to have beneficial effects on lung cancer (98), colorectal cancer (99), glioblastoma (100), and breast cancer (101).

After using cardiac glycosides on breast tumor cells, CRT exposure on the cell surface and extracellular release of ICD-related DAMPs such as HSP70/90, ATP, and HMGB1 increased. It was demonstrated that activation of the PERK-elF2α signaling pathway induced ER stress response, leading to CRT exposure. After administration of cardiac glycosides, CD80 and 86 expression increased and acted as a co-stimulatory signal to activate cytotoxic T lymphocytes. In contrast, IL-2 and IFN-γ levels increased significantly, suggesting enhanced antitumor immune effects of Th1 and NK cells and dose-dependent changes in cytotoxic T lymphocytes (66).

In conclusion, cardiac glycosides can induce ICD responses in tumor cells, improve the TME, inhibit tumor cell development, and promote apoptosis, and are highly promising ICD inducers.

#### 3.1.5 Protein kinase inhibitor-based inducers

Protein kinase inhibitors are enzymes that catalyze protein phosphorylation and play an important role in gene expression. They are classified as serine/threonine protein kinase inhibitors and tyrosine-protein kinase inhibitors, and alterations in their activity are associated with the development of several malignancies (102–104). Tyrosine protein kinase inhibitors inhibit tumor cell growth and promote apoptosis by inhibiting cell signaling. This class of protein kinase inhibitors includes sunitinib, crizotinib, ceritinib and cabozantinib. These inhibitors act on tumor cells to induce all features of the ICD response, including ATP secretion, HMGB1 release, CRT exposure, and other related DAMPs. Different doses of these inhibitors have different effects on tumor cells, and high doses of tyrosine kinase inhibitors inhibit the transcriptional process of DNA and affect the growth of tumor cells (71, 75, 105).

Erlotinib is an epidermal growth factor receptor tyrosine kinase inhibitor, and its long-term application leads to the development of EGFRI resistance, which is characterized by an increase in cellular autophagic flux. Its cytoprotective autophagic flux is inhibited by the selective tyrosine kinase inhibitor bemcentinib, which blocks the cloning of drug-resistant tumor cells and further induces the development of ICD response in tumor cells. Concurrently, proinflammatory cytokine release was increased, cytotoxic T lymphocytes were activated and proliferated, and antitumor immune effects were enhanced with significant anticancer effects (74). The joint action of the two tyrosine kinase inhibitors resolved the poor anticancer effects of drug-resistant tumor cells. Surprisingly, the immune TME was changed, further enhancing the antitumor immune effect, which provides a theoretical basis for the clinical treatment of drug-resistant tumors.

PLK1 and CDK12/13 are both serine/threonine protein kinases, and the application of inhibitors of these two protein kinases in tumor cells revealed a dose-dependent increase in CRT levels as well as an increased release of DAMPs, such as ATP and HMGB1, and an ICD response. The mechanism of ICD response induced by this type of protein kinase lies in the phosphorylation of IRE1 and EIF2α, activation of the PERK signaling pathway, stress response of the endoplasmic reticulum, and alteration of the immune TME, including the maturation/activation of DCs, increased release of proinflammatory cytokines, enhanced expression of IFN-γ, and increased number of cytotoxic T lymphocytes. These changes significantly enhanced the antitumor immune effect and antitumor efficacy (73, 76).

In conclusion, protein kinase inhibitors are highly promising ICD inducers that can improve the TME, promote tumor cell death, and significantly improve antitumor efficacy of tumor therapy.

#### 3.1.6 Inducers for targeting tumorigenesis-related substances

Proteins and substances related to protein synthesis are involved in the metabolic pathways of tumor cells. Furthermore, they play a coordinating role in the growth of tumor cells by regulating their metabolism, immunity, and other functions (106, 107).

Arginine is a critical amino acid in protein synthesis, and its degradation promotes the death of tumor cells (108). Arginine deiminase (ADI) is the most common arginine degradation enzyme, and it can form ADI-PEG 20 when combined with 20 kDa polyethylene glycol. After ADI-PEG 20 was applied to tumor cells, the intracellular mTOR signaling pathway was activated, which plays an important role in cell death by regulating cellular autophagy. Concurrently, ADI-PEG 20 inhibited the cell cycle in tumor cells and severely disrupted intracellular Ca2^+^ homeostasis and mitochondrial membrane depolarization. Under the effect of multiple factors, large numbers of tumor cells died under the action of ADI-PEG 20 (77).

BET proteins have been shown to control the expression of cancer-related genes, indicating that they play an important role in tumor cytogenesis (109). Drugs targeting BET proteins, including BETd246 and BETd260, have potent anticancer activities. In colorectal tumor cells, drugs targeting BET proteins activated the apoptotic signaling pathway caspase3/8/9. Simultaneously, DR5 was also activated. Activation of the DR5 signaling pathway mainly originated from the endoplasmic reticulum stress response, CHOP signaling activation, and phosphorylation of EIF2α. DR5 signaling pathway-mediated apoptosis occurred in colorectal tumor cells. During apoptogenesis, colorectal tumor cells expose and release many DAMPs associated with the ICD response. Moreover, the TME was greatly improved, cytotoxic T lymphocyte infiltration was increased, immunomodulatory T cells were significantly reduced, and antitumor immune function was greatly improved (78).

#### 3.1.7 Mitochondrial uncoupler-based inducers

The growth of tumor cells depends on the function of the mitochondria, which produce tumor-associated metabolites through aerobic glycolysis, causing adverse effects on the body (110). Mitochondrial metabolism is the basis of tumor cell activity and plays an important role in the growth and metastasis of tumor cells (111, 112). The role of mitochondrial uncouplers is to transfer protons from the inner membrane of the mitochondria to the matrix through a pathway unrelated to ATP synthase so that nutrient metabolism is not linked to ATP production (113). Several mitochondrial uncouplers have been shown to significantly affect tumor cell growth, including classical mitochondrial uncouplers, such as niclosamide (114), nitazoxanide (115), and oxyclozanide (116). Mitochondrial uncouplers usually impede tumor cell growth by promoting mitochondrial autophagy dysfunction, activating the AMPK signaling pathway to inhibit mTOR signaling, inhibiting the Wnt signaling pathway, and inducing massive ROS production (117).

2,5-dichloro-N-(4-nitronaphthalen-1-yl) benzenesulfonamide (Y3) is a mitochondrial uncoupler that acts on Y3 in epithelial ovarian cancer (EOC). Y3 activated CHOP signaling, EIF2α phosphorylation, and the endoplasmic reticulum stress response in tumor cells. Concurrently, the AMPK signaling pathway was also activated. Activation of this signaling pathway inhibited the growth of tumor cells and promoted apoptosis. During apoptosis, ATP, HMGB1, and other ICD-related DAMPs were released into tumor cells. In the TME, the release of these DAMPs stimulated the release of proinflammatory cytokines, the activation of cytotoxic T lymphocytes, and the complete change in the suppressive TME. The body’s antitumor immune response was further enhanced, and ICD of tumor cells occurred (79).

#### 3.1.8 Other chemical inducers

In addition to the inducers mentioned above, several novel chemical agents have been recently shown to induce ICD responses in tumor cells in basic experiments. These new ICD inducers include resveratrol (118), non-steroidal anti-inflammatory drugs (86), PKHB1 (88), and belantamab mafodotin (80).

Resveratrol and non-steroidal anti-inflammatory drugs are widely used in various applications, as anti-inflammatory, anti-microbial, and anti-viral drugs (119, 120). In recent years, experiments have also demonstrated the ability of these two classes of drugs to exert antitumor effects (121, 122). Resveratrol is synthesized in grape leaves and skin and is an antitoxin produced when the plant is irritated. When resveratrol was applied to ovarian tumor cells, the proliferation of ovarian tumor cells was inhibited and apoptosis occurred. During apoptosis, CRT, HMGB1, ATP, and other ICD-related DAMPs were released in large quantities or expressed on the tumor surface, and ICD occurred. Concurrently, the TME was substantially improved, and DC maturation and activation occurred, accompanied by a significant increase in cytotoxic T lymphocytes and a large release of proinflammatory cytokines. The antitumor immune response was further enhanced. However, the mechanism of resveratrol-induced ICD response in tumors remains unclear and requires further investigation (118).

In the same way as in response to resveratrol, a similar ICD response occurred after the application of NSAIDs on colorectal tumor cells, and the proliferation of colorectal tumor cells was inhibited. This is due to the severe endoplasmic reticulum stress response of NSAIDs on tumor cells, activation of intracellular PERK signaling, phosphorylation of EIF2α, and enhancement of the DR5 signaling pathway mediated by this pathway. Simultaneously, intracellular pro-apoptotic caspase-8 and BID proteins were significantly increased. Under the combined effect of multiple triggers, the antitumor immune response was enhanced, and many tumor cells underwent ICD (86).

In addition to these two drugs, PKHB1 is a newly discovered ICD inducer. PKHB1 is a stable agonist peptide that induces tumor cell death associated with CD47 activation. Moreover, its action on tumor cells induces alterations in mitochondrial structure, Ca^2+^ accumulation, and calcium-dependent cell death. In addition, it increased the number of cytotoxic T lymphocytes, DC activation and maturation, and decreased immunosuppressive cells such as Treg cells, promoting tumor cell death (87, 88).

Surprisingly, in response to the BCMA-targeted antibody-drug conjugate GSK2857916 under the effect of EIF2α phosphorylation and PERK signaling pathway activation, the endoplasmic reticulum of tumor cells underwent a stress response and *in vivo* DC activation and maturation. The number of cytotoxic T lymphocytes increased, but that of Treg cells in the tumor increased significantly. This is contrary to the expectation of an enhanced antitumor immune effect, which may be related to the prevention of autoimmunity and avoidance of adverse effects (80).

In conclusion, all of these novel ICD inducers showed standard features of ICD responses after acting on tumor cells, including CRT exposure and extracellular release of DAMPs such as ATP and HMGB1. This inhibited the growth of tumor cells by altering the immune TME, enhancing the recruitment of immune cells and phagocytosis of tumor cells, and significantly enhances the antitumor immune response by promoting the death of a large number of tumor cells, significantly improving the effect of antitumor therapy.

### 3.2 Physical sources of ICD induction

Patients undergoing chemotherapy can experience severe side effects and intolerable pain, and the use of physical methods to induce an ICD response can effectively mitigate these side effects (123). In recent years, the use of physical methods to induce ICD in treating patients with tumors has become a hot research topic. Physical methods demonstrated to induce ICD in tumor cells include photochemotherapy, thermotherapy, high hydrostatic pressure, and plasma irradiation (124–127). The induction mechanisms of these physical methods and their effects is shown in **Table 2**.

**Table 2:** Physical sources of ICD induction.

Inducer classification	Induction method	Cell lines	Induction mechanism	Induction effect	References
Type I	Infrared light heat therapy	B16F10	Increased release of DAMPs such as ATP, HMGB1, and heat shock proteins; cell injury	ICD induction; massive T cell proliferation; significant upregulation of CD80, CD86, MHC-II, CD40; DC maturation; inflammatory cytokine release	(128)
Type I	Oncology treatment field	LLC-1, CT-26, HepG2, H520, MOSE-L	EIF 2α phosphorylation; ER stress response; disruption of cytokinesis; enhanced autophagic response; CRT exposure; ATP and HMGB1 release	ICD induction; *in vitro* DC maturation; *in vivo* leukocyte recruitment; increased IFN-γ production; enhanced antitumor immune function	(129)
Type I	Radiation therapy	PC3; DU145; LNCAP	Increased release of DAMPs such as ATP and HMGB1.	ICD induction; DC maturation; elevated GM-CSF levels; inflammatory response	(130)
Type II	High hydrostatic pressure	OV-90; CT-26; ALL; LNCAP	ER stress response based on ROS stimulation; EIF 2α phosphorylation; PERK signaling pathway activation; caspase-2, 3, 8 activation, CRT exposure; HSP protein expression; ATP, HMGB1, and other DAMPs release,	DC maturation; proliferation of antigen-specific T cells; ICD induction; significant upregulation of CD83, CD86, and HLA-DR; production of inflammatory substances such as IL-1, IL-6, and TNF-α	(124, 131)
Type II	CAP Cold atmospheric plasma	HCT-116; BCPAP; CT26; A431	CRT exposure; release of DAMPs such as HMGB1, heat shock proteins; activation of MAPK and NF-κB pathways	ICD induction; G-CSF elevation; IL-4 decrease; IL-1, IL-6, and TNF-α inflammatory factor release; enhanced antitumor immune function	(125, 132)

LLC-1, Lewis lung cancer; CT-26, mouse colon cancer cells; HepG2, human liver cancer cells; H520, human lung squamous cell carcinoma cells; MOSE-L, mouse ovarian surface epithelial cells; PC3, DU145, LNCAP, human prostate cancer cells; ALL, acute lymphoblastic leukemia; B16F10, mouse melanoma cells; HCT-116, human colorectal cancer cells; BCPAP, human thyroid cancer papillary cells; A431, human epidermoid cancer cells.

#### 3.2.1 Radiotherapy

Conventional radiation therapy is one of the most commonly used treatments for tumors with a long history. It works by applying small doses of continuous radiation to the site of tumorigenesis, inducing DNA double-strand breaks and other damage to eliminate cells in specific areas of the body. Radiation affects tumor and normal cells, resulting in growth restriction and apoptosis of these cells, thus achieving the goal of eliminating tumor cells. The aim is to eliminate tumor cells; however, there are many side effects (133). Currently, radiation therapy is widely used and has achieved good efficacy in the clinical treatment of prostate (134), breast (135), cervical (136), and other cancers.

Stereotactic body radiation therapy (SBRT) is a new type of radiation therapy. Compared to conventional radiation therapy, this treatment method provides a higher single dose over a shorter total treatment time. Moreover, the precise positioning of radiation sites with the help of computer images can effectively reduce the damage caused to the surrounding tissues and cells, thus reducing the side effects and significantly relieving the pain that patients with tumors endure during physical therapy. It also improves the efficacy of tumor treatment (137, 138).

After conventional radiation and stereotactic body radiation therapies, the expression of DAMPs related to ICD response, such as CRT, HSP, and HMGB1, increased in tumor cells. Moreover, cytotoxic T lymphocytes proliferated, immunosuppressive cells such as Tregs were suppressed, proinflammatory cytokine expression increased, and TAA expression related to immune evasion increased, suggesting that immune evasion was suppressed. Furthermore, these changes in immunoregulatory genes significantly altered the immune TME, promoted tumor cell death, and improved the efficacy of immune therapy (61, 130, 139).

In conclusion, the conventional and new stereotactic body radiation therapies are associated with the induction of immunomodulatory genes, which cause an immune response and induce immunogenic death of tumor cells.

#### 3.2.2 High hydrostatic pressure

Application of high hydrostatic pressure (HHP) is a novel treatment method in clinical oncology. The average hydrostatic pressure on earth is 40 MPa. Different pressure ranges have different effects on biomolecules, cellular processes, and cell viability. HHP is divided into physiological HHP (<100 MPa) and non-physiological HHP (>100 MPa) (140). Pressures above normal levels can have different effects on cells; for example, hydrostatic pressure <100 MPa slightly affects cell morphology but is not sufficient to cause cell death, that between 100–150 MPa causes cell death in mice, and that >200 MPa affects the viability of human cells, and even causes apoptosis, depending on the type and sensitivity of the cells; At a hydrostatic pressure of >300 MPa, most cells are necrotic. Based on this information, researchers have achieved a pro-apoptotic effect on tumor cells by adjusting hydrostatic pressure, which significantly improved the efficacy of antitumor therapy (140, 141).

Preclinical studies have used hydrostatic pressure therapy for ovarian, colon, and prostate tumor cells. When these tumor cells were treated with HHP, following changes occurred: excessive ROS was produced, peroxidase activity increased, the PERK signaling pathway was activated, EIF2α was phosphorylated, caspase-2, 3, 8, and 9 were rapidly activated, and endoplasmic reticulum stress response occurred. Concurrently, ICD-related DAMPs, including CRT, heat shock proteins expressed on the tumor surface, HMGB1, and ATP, were released in large quantities. Moreover, the maturation and activation of DCs, significant upregulation of CD83 and 86, proliferation of cytotoxic T lymphocytes, release of proinflammatory cytokines in large quantities occurred and immune TME were significantly changed; ultimately, and the immune response was enhanced (124, 131).

In clinical trials, HHP has been shown to induce the release of many ICD-associated DAMPs during tumor cell development, further promoting DC maturation. In addition, HHP kills tumor cells while retaining many tumor-associated-specific antigens in the TME. These characteristics of HHP therapy indicate its great potential for whole-cell tumor or DC-based tumor vaccine preparation (141). Whole-cell tumor vaccines prepared using HHP therapy have shown to be effective in clinical trials for multiple myeloma (142) and renal cell carcinoma (143). In addition, the use of HHP therapy to prepare DC-based tumor vaccines is advancing. DC-based tumor vaccines also achieved positive outcomes in clinical trials, including those in prostate and ovarian cancers (144).

In conclusion, cells treated with HHP are immunogenic, *in vivo*, and the core of this ICD lies in the activation of the ROS-PERK-EIF2α phosphorylation–caspase signaling pathway, which is a reliable and effective physical method to induce ICD response in tumor cells.

#### 3.2.3 Near-infrared light-mediated thermotherapy

Near-infrared light-mediated thermotherapy can damage tumor cells. Thermotherapy achieves cell-killing effects through mechanisms of action such as destabilization of the cytoskeleton and effect on cell cycle progression. At high temperatures (>44°C), cells under the influence of thermal mechanisms of action undergo extensive cell damage and cell death, which is usually induced at moderate to high temperatures (41–42°C) (145). In a study using NIR light thermotherapy (41.5°C, 1 h) on melanoma cells, the treatment resulted in the release of ICD-related DAMPs, such as tumor cell HSP and HMGB1, maturation/activation of DC, and increased release of proinflammatory cytokines, which induced a robust antitumor immune response. Under the immune effect of the ICD response, tumor cells die in large numbers (128). The mechanism by which NIR light-mediated thermotherapy can significantly reduce tumor resistance and induce an immune response proves that this approach is a novel and effective inducer of the ICD response, which can result in a better prognosis for patients with tumors receiving this treatment.

#### 3.2.4 Plasma irradiation therapy

Cold atmospheric plasma, an ionized gas operating at room temperature, is a promising new physical method for inducing tumor cell ICD reactions; however, its specific anticancer mechanism is still unclear. Nevertheless, some studies have shown that cold atmospheric plasma can activate intracellular oxidative stress signals, causing DNA double-strand breaks and thus apoptosis (146, 147). This cold-atmosphere plasma treatment method is currently used for bladder (148), cervical (149), esophageal (150), and prostate cancer (151). It effectively exerts anticancer activity and reduces host mortality rate and incidence of side effects. In a study of the effect of cold-atmosphere plasma treatment on colorectal cancer cells, cold-atmosphere plasma induced a robust antitumor immune response in the host by activating the MAPK and NF-κB signaling pathways. Furthermore, tumor cells were significantly externalized by CRT and HSP during the cell death process, and ICD-related DAMPs such as HMGB1 were released in large quantities, suggesting that tumor cells in the apoptotic process with significant immunogenic features underwent ICD. In contrast, the TME in the host was altered, with the massive proliferation of cytotoxic T lymphocytes, massive recruitment of immune-related cells, massive release of proinflammatory cytokines, maturation and activation of DCs, and further strengthening of the antitumor immune response in the host (125, 132). In conclusion, cold atmospheric plasma therapy is a highly effective ICD inducer with potent anticancer activity and has broad prospects in the clinical treatment of tumors.

#### 3.2.5 Tumor treating fields

Tumor treating fields (TTFields) is a non-invasive tumor treatment method with a low intensity (1–3 v/cm) alternating electric field in the medium frequency range (100–300 kHz). The mechanism of action involves interfering with cell proliferation and promoting cell death by interfering with microtubule assembly, exerting different directional forces to induce antimitotic effects, and triggering the formation of abnormal structures during spindle formation (152, 153). Recent studies have shown that the mechanism of action of TTFields is not only to interfere with mitosis to inhibit tumor cell proliferation but also to disrupt many biological processes, including blocking DNA repair, and increasing cell permeability, thereby promoting cell death (154). The application of TTFields can lead to phosphorylation of EIF2α, an important marker for the onset of the endoplasmic reticulum stress response, while CRT is exposed on the cell surface. The action of TTFields leads to enhanced autophagy, resulting in the release of DAMPs such as ATP and HMGB1, suggesting the occurrence of ICD response in tumor cells, along with DC maturation and activation, massive recruitment of immune-related cells *in vivo*, and enhanced antitumor immune effects. After the combined effect of TTFields therapy and anti-PD-1, cytotoxic T lymphocytes proliferated in large numbers, IFN-γ was released in large amounts, the TME was significantly improved, and antitumor immune function was further enhanced (129).

In conclusion, the TTFields therapy is a new type of ICD inducer, and its anticancer activity has been confirmed in preclinical experiments. It is positive to note that the U.S. Food and Drug Administration (FDA) approved the use of TTFields for the clinical treatment of specific tumors in 2011 (155). These tumors include newly diagnosed or recurrent glioblastoma and malignant pleural mesothelioma, and TTFields have significantly prolonged the survival of patients with these tumors (153). In addition, the clinical outcomes of TTFields did not differ significantly from those of chemotherapy. However, TTFields have minimal toxicity and a better prognosis, providing a new option for many patients with tumors who cannot tolerate chemotherapy (156). Clinical studies of TTFields for other solid tumors are underway in addition to those on glioblastoma and malignant pleural mesothelioma. These include pancreatic (157), ovarian (158), non-small cell type lung cancer, and non-small cell type lung cancer with brain metastases (159). It is encouraging to note that the results of these clinical studies have been positive, and it is expected that TTFields will have a promising future in the clinical treatment of tumors and bring hope to many patients with tumors.

### 3.3 Pathogen-derived ICD inducers

Oncological patients often experience tumor recurrence and metastatic complications after receiving conventional chemotherapy or physical therapy due to increased tolerance to the treatment method (160). The advent of pathogen derivative-mediated oncological therapies offers new options for tumor treatment. Pathogen derivatives have been shown to have positive effects on tumor killing and inhibition of tumor metastases when used alone or in combination therapy (161, 162). Several pathogen-derived substances, including lysozyme virus, mitomycin C, and Alternol, have been shown to induce ICD in tumor cells (163–165). The induction mechanisms and effects of pathogen-derived ICD inducers are shown in **Table 3**.

**Table 3:** Pathogen-derived ICD inducers.

Inducer classification	Inducer	Cell lines	Induction mechanism	Induction effect	References
Type II	Alternol	LNCaP; 22RV1; PC-3; RM-1	ER stress response based on ROS stimulation; CRT exposure; release of DAMPs such as ATP, HMGB1; EIF2α phosphorylation.	Increased CD80 and CD86; ICD induction; decreased immunosuppressive regulatory T cells (Tregs); increased expression of proinflammatory cytokines	(163)
Type II	Mitomycin C	5637	Mitochondrial metabolic reprogramming based on oxidative phosphorylation; CRT exposure; release of DAMPs such as ATP, HMGB1; activation of PERK, IRE signaling pathways	DC maturation; ICD induction; increased expression of proinflammatory cytokines; increased expression of CD80 and CD86	(164)
Type II	Oncolytic virus	A549; HOS	CRT exposure; release of DAMPs such as ATP, HMGB1; irreversible cellular damage.	ICD induction; cytotoxic T lymphocyte recruitment; increased expression of CD86 and CD80; DC maturation; increased secretion of proinflammatory cytokines	(166, 167)

A549, human lung epithelial carcinoma cells; LNCaP, 22RV1, PC-3, human prostate cancer cells; RM-1, mouse prostate cancer cells; 5637, human bladder cancer cells; HOS, human osteosarcoma cells.

#### 3.3.1 Virus-derived ICD inducers

Biological therapies have long been effective in clinical cancer therapy. Among these the most typical is tumor oncolytic virus therapy, which can selectively replicate in tumor cells and kill them through a lysis-reactions with minimal impact on normal cells, effectively reducing the occurrence of adverse effects. These drugs have passed multiple rounds of clinical trials and have been applied in many clinical treatments for tumors, significantly improving the overall survival rate of patients (168–170).

Oncolytic viruses with anticancer activity have been identified, including wild-type adenovirus, Semliki Forest virus (SFV), vaccinia virus, and oncolytic Newcastle disease virus, which release immunostimulatory molecules during cell lysis, thereby enhancing the immune response. Additionally, all of these oncolytic viruses increased the release of extracellular ATP and HMGB1 and the surface exposure of DAMPs such as CRT and heat shock proteins during the action, suggesting that tumor cell ICD response occurred during the action of these viruses, further enhancing the antitumor immune effect (165, 171). The process of antitumor immunity varies among oncolytic viruses. Tumor cells treated with wild-type adenovirus have enhanced cellular autophagy, which induces IFN-γ secretion through enhanced STING signaling and triggers the antitumor response of cytotoxic T lymphocytes while promoting APC recruitment and phagocytosis. The antitumor immune response was enhanced by DC activation and maturation in tumor cells acted upon by the Semliki Forest virus, which produced Th1 and proinflammatory cytokines (166). In the presence of oncolytic Newcastle disease virus (NDV) in lung cancer cells, DC activation and maturation occurred, and proinflammatory cytokines, NK cells, and cytotoxic T lymphocytes were substantially increased. This ICD was associated with autophagy-related genes in lung cancer cells, independent of the apoptotic process mediated by cysteine aspartate-specific proteases (caspases) and cellular necrosis (165).

#### 3.3.2 Microflora-derived ICD inducers

The treatment with microbial derivatives is also an integral part of the biological treatment of tumor cells. Mitomycin C, a compound extracted from *Streptococcus* spp., exhibits genotoxic and anticancer activities. The primary mechanism involves inhibition of DNA, RNA, and protein synthesis (172). The anticancer process of mitomycin induces ICD, increases oxidative phosphorylation through metabolic reprogramming, alters cellular mitochondrial permeability, promotes inflammatory cytokines and DC activation and maturation, and enhances antitumor immunity (164).

Alternol is a newly discovered compound found in microbial mutagenic strains. Like most ICD inducers, this compound triggers endoplasmic reticulum stress response through ROS production, which in turn causes the ICD response and massive release of ICD-related DAMPs. After Alternol acts on tumor cells, cytotoxic T lymphocytes increase, DC activation and maturation occurs, Tregs are suppressed, and the immune TME *in vivo* is altered, promoting occurrence of an antitumor immune response (163).

The emergence of this series of biological therapies has dramatically enriched the options available for cancer treatment and has profound implications for the overall survival of patients.

## 4 Combined effect of ICD inducers

In the process of tumor treatment, the long-term application of a single treatment method can lead to poor antitumor efficacy or tumor cell resistance. In addition, using a single treatment method can reduce the survival of patients with tumors by subjecting them to different side effects due to different factors, such as high doses and longer treatment periods. Combination therapy is a method to improve the efficacy of tumor treatment and reduce the possibility of drug-resistant tumor cells. Simultaneously, combination therapy can precisely control the dose of each chemotherapy drug and the treatment period, which can significantly reduce the side effects that patients with tumors experience during treatment and improve the survival of these patients (173, 174).

### 4.1 Combined use of multiple chemical inducers

STAT3 is a transcription factor with many vital functions in various cell types, including the regulation of cell proliferation, differentiation, death, angiogenesis, inflammation, and immune response. Abnormal STAT3 activity causes tumors to release large amounts of immunosuppressive factors (175, 176). STATTIC, a STAT3 inhibitor, can selectively inhibit STAT3 dimerization, activation, and nuclear translocation, induce STAT3-dependent apoptosis in tumor cells, and significantly reduce tumor growth (177).

In a study on the combined application of oncolytic Newcastle disease virus (NDV) and STATTIC in prostate cancer cells, ICD-related DAMPs such as HMGB1, heat shock protein, and ATP were released in large amounts after combined treatment compared with those released after the application of oncolytic virus alone. The expression of VEGF, as well as angiogenesis, was inhibited in prostate cancer cells after the action of STATTIC. In conclusion, inhibition enhanced NDV-induced oncolytic death in prostate tumor cells (167). In contrast to STAT3 in prostate tumors, STAT3 inhibition decreased the effect of NDV on melanoma cells, further reducing the release of ICD-associated DAMPs (178). Importantly, NDV is used to treat different tumor types have different effects when the context of inhibiting STAT3 expression. This difference may depend on the different tumor origins, and the underlying mechanisms remain to be explored.

In addition to oncolytic viral agents, STATTIC, combined with adriamycin, can synergistically fight tumors with higher secretion levels of ICD-related DAMPs. STATTIC can significantly enhance the effect of chemotherapeutic agents in reversing tumor immunosuppression and producing a strong antitumor immune response (179).

Cisplatin is one of the most commonly used platinum compounds for tumor chemotherapy. Studies have shown that cisplatin alone does not induce an ICD response in tumor cells or the onset of the endoplasmic reticulum stress response (180). The combination of cisplatin and 5-fluorouracil also induced a significant release of HMGB1, DC maturation/activation, upregulation of CD80 and CD86 in tumor cell ICD response, and further enhancement of the host antitumor immune response with a favorable prognosis (72). These studies suggest that combination therapy restored the immunogenicity of cisplatin and promoted the development of ICD response in tumor cells.

Paclitaxel analogs are also commonly used in oncology chemotherapy, and this class of drugs has substantial side effects (180). A study applied paclitaxel analogs in combination with sunitinib for breast cancer treatment, and sunitinib was able to reduce neovascularization and alter the immunosuppressive TME. Compared with the application of paclitaxel analogs alone, the combination resulted in complete release of HMGB1 from the nucleus and a significant increase in CRT exposure on the cell surface. Further ICD response in tumor cells was enhanced, DC maturation/activation occurred, Tregs and other immunosuppressive cells were reduced, and tumor immunogenicity was further enhanced. Additionally, the combined effect of the two drugs reduced the dosage of paclitaxel when used as a single drug, the side effects suffered by patients were significantly reduced, and the survival rate of hosts with tumors was significantly improved (71).

### 4.2 Combined use of chemical inducers and physical induction methods

Combining chemical and physical methods to induce an ICD response in tumor cells can improve the effectiveness of anticancer treatment and significantly reduce treatment duration compared to the application of chemotherapy alone. In the process of physically inducing an ICD response in tumor cells, the presence of unique instruments makes it possible to precisely locate the site of tumorigenesis and to apply chemical agents to specific areas. Patients benefit from the use of precise treatment during the combined treatment, the pain and discomfort will be significantly reduced, and the patient’s quality of life will be maintained while the efficacy of the treatment is improved. Owing to the benefits of combination therapy, this treatment method has been used in many clinical treatments (181).

Radiotherapy using X-rays is one of the most widely used methods of physical tumor therapy, and the use of radiation therapy alone has a damaging effect on tumor cells, but this effect is not significant. A study combined classical anticancer chemotherapeutic drugs, such as oxaliplatin, 5-fluorouracil, cisplatin, and adriamycin, with radiation therapy. Compared to the application of radiation therapy alone, the combined action showed a significant increase in CRT surface exposure, a large release of ICD-related DAMPs such as HMGB1 and ATP, and significant proliferation of cytotoxic T lymphocytes, and recruitment of immune-related cells in large numbers. The tumor cell ICD response was further enhanced. Based on the antitumor mechanism of 5-fluorouracil, the cell cycle of tumor cells is blocked, whereas in combination with radiation therapy, the response rate of immunotherapy is increased, the growth and metastasis of the primary tumor are inhibited, and the patient’s prognosis is improved (61, 182–184). Moreover, radiation therapy combined with chemotherapy upregulated PD-1 expression in tumor cells, anti-PD-1 antibodies enhanced antitumor immune activity, and the host antitumor immune effect was further improved by adding anti-PD-1 antibodies after combination therapy (185, 186).

In addition to radiation therapy, photodynamic therapy (PDT) for tumors is used in a large number of clinical treatments. Some studies combined PDT with oxaliplatin. The combined therapy acts on the tumor cells by increasing ROS levels and enhancing cytotoxic effects. Compared with oxaliplatin treatment alone, CRT exposure and HMGB1 increased in large numbers, inducing cellular ICD responses. Under DC maturation/activation, IFN-γ mass expression, and cytotoxic T lymphocytes proliferation, tumor cells underwent immunogenic apoptosis and necrosis, and tumor growth was inhibited. Additionally, high levels of perforin and granzyme had killing effects on tumor cells, and the tumor immune effect was further improved (187).

In conclusion, the combined use of physical and chemical methods can enhance the release of each DAMP during ICD, change the host immune TME, improve the efficacy of anticancer treatment, and reduce side effects during the treatment of patients with tumors. The clinical application of this combined therapy provides a reliable option for many patients.

### 4.3 Combined application of multiple physical induction methods

Melanoma is a tumor that can tolerate radiation, and thus, radiation therapy alone is ineffective in treating melanoma. One study used a combination of heat and radiation therapies to treat melanoma cells. Compared with radiation therapy alone, the combined effect of heat and radiation therapy on tumor cells induced ICD response, increased the release of ICD-related DAMPs, altered the immunosuppressive TME, and further enhanced the host antitumor immune effect (188).

### 4.4 Hazards of incorrect combination of inducers

In addition to promoting apoptosis of tumor cells, an inappropriate combination of various therapies can further accelerate tumor cell development and deterioration of the patient’s condition. Mitoxantrone-induced ICD response in tumor cells requires proteasome activation. However, the combination with proteasome inhibitors significantly attenuates the release of ICD-related DAMPs, suggesting that the mitoxantrone-induced ICD response inhibits tumor cell growth and worsens the patient’s condition (69). Similarly, the combination of 5-fluorouracil and MTIF2 also leads to deterioration of the patient’s condition. Downregulation of MTIF2 affects tumor cell proliferation and migration. In addition, the drug resistance of tumor cells is weakened, the overexpression of MTIF2 and ICD-related DAMPs is significantly reduced, DC maturation/activation is impaired, tumor immunosuppression occurs, and tumor cells rapidly proliferate and patients have a poor prognosis (65).

In conclusion, combination therapy is a future trend in tumor treatment. Whether it promotes or suppresses antitumor immune effects, combination therapy provides a detailed theoretical basis for clinical treatment. The widespread use of combination therapy can benefit tens of thousands of patients with tumors.

## 5 New techniques for ICD induction

The ICD response can be induced by chemicals, pathogen derivatives, and physical methods that promote apoptosis and inhibit tumor cell development. However, these conventional ICD-inducing agents have different limitations and challenges in their practical clinical use, including their safety and efficacy against different tumors (189). With the advancement of technology, new techniques to induce ICD have emerged that can effectively solve these problems. The induction mechanisms and effects of these new techniques are summarized in **Table 4**.

**Table 4:** New techniques for inducing immunogenic cell death in tumor cells.

Induction method		Induction medium	Cell line	Characteristics and mechanism	Effectiveness	References
Photodynamic therapy	Conventional photodynamic therapy	Conventional photosensitizers such as porphyrins (PZ I/III) and 8-methoxypsoralen (8-MOP)	B16-OVA; MCA205	Activation of PERK signaling pathway; eIF2α phosphorylation; ROS-related endoplasmic reticulum-based stress response; CRT translocation exposure; ATP, HMGB1, and other DAMPs release	ICD response onset; cytotoxic T lymphocyte recruitment; DC maturation/activation; decreased immunosuppressive regulatory T cells; increased CD80 and 86 expression	(190, 191)
	Infrared light-mediated targeted therapy	IRDye700DX and other targeted photosensitizers	NIH3T3; TE4; OE19	Targeted recognition of tumor cell surface-specific monoclonal antibodies; irreversible cell damage; CRT and HSP translocation exposure; release of DAMPs such as ATP and HMGB1	ICD response onset; cytotoxic T lymphocyte recruitment; DC maturation/activation; immunosuppressive regulatory T cell reduction	(192, 193)
Nano-pulse stimulation technology		——	4T1	Alteration of tumor cell membrane permeability; rearrangement of Ca2^+^; endoplasmic reticulum stress response; CRT, HSP translocation exposure; release of DAMPs such as ATP, HMGB1	ICD response onset; DC maturation/activation; reduction in immunosuppressive regulatory T cells	(194)
Carrier-mediated ICD induction	Exogenous vector-mediated induction of ICD	CN@PHF	4T1	Under neutral conditions, the carrier has high drug loading capacity, stability, and targeting. Also, the drug accumulates faster in the tumor and has a high concentration.	ICD response onset; DC maturation/activation; immunosuppressive regulatory T cell reduction; massive release of proinflammatory cells; proliferation of cytotoxic T lymphocytes	(195)
		HPMA copolymer	4T1; MDA- MB231; CT26; Hepa1-6; B16	Good biocompatibility, good water solubility, non-toxic, passive targeting; inhibition of PI3K signaling pathway; CRT translocation exposure; ATP, HMGB1, and other DAMPs release	ICD response onset; immunosuppressive regulatory T cell reduction; massive release of proinflammatory cytokines; proliferation of cytotoxic T lymphocytes	(196, 197)
	Endogenous vector-mediated induction of ICD	Cell membrane carrier	Mouse lung cancer cell line	Extremely stable; high biocompatibility; tumor homing properties; CRT translocation exposure; release of DAMPs such as ATP, HMGB1	ICD response onset; DC maturation/activation; immunosuppressive regulatory T cell reduction; cytotoxic T lymphocyte proliferation	(198, 199)
		Exosomes	BM-MSC	Contains biologically active substances; low immunogenicity, able to avoid accidental phagocytosis; CRT translocation exposure; release of DAMPs such as ATP and HMGB1	ICD response onset; massive release of proinflammatory cytokines; proliferation of cytotoxic T lymphocytes	(200, 201)
		Liposomes	MCF7; CT26; CT26-Luc; PC3; B16	ROS-based endoplasmic reticulum stress response; CRT translocation exposure; release of DAMPs such as ATP and HMGB1; ability to load hydrophilic/hydrophobic drugs	ICD response onset; cytotoxic T lymphocyte proliferation	(202)

MCA205, mouse fibrosarcoma; B16, mouse melanoma; TE4, HER2-positive squamous cell carcinoma; OE19, HER2-positive adenocarcinoma; 4T1, mouse triple-negative breast cancer cell line; MDA-MB231, human triple-negative breast cancer cell line; Hepa1-6, mouse hepatocellular carcinoma cell line; MCF, 7 human breast cancer; CT26, Luc-luciferase labeled mouse colon cancer; PC3, human prostate cancer; NIH3T3, mouse fibroblast cell line; MDA-MB-23, human breast cancer cells; BM-MSC, mouse bone marrow mesenchymal stem cells.

### 5.1 Photodynamic therapy

**Figure 3:**
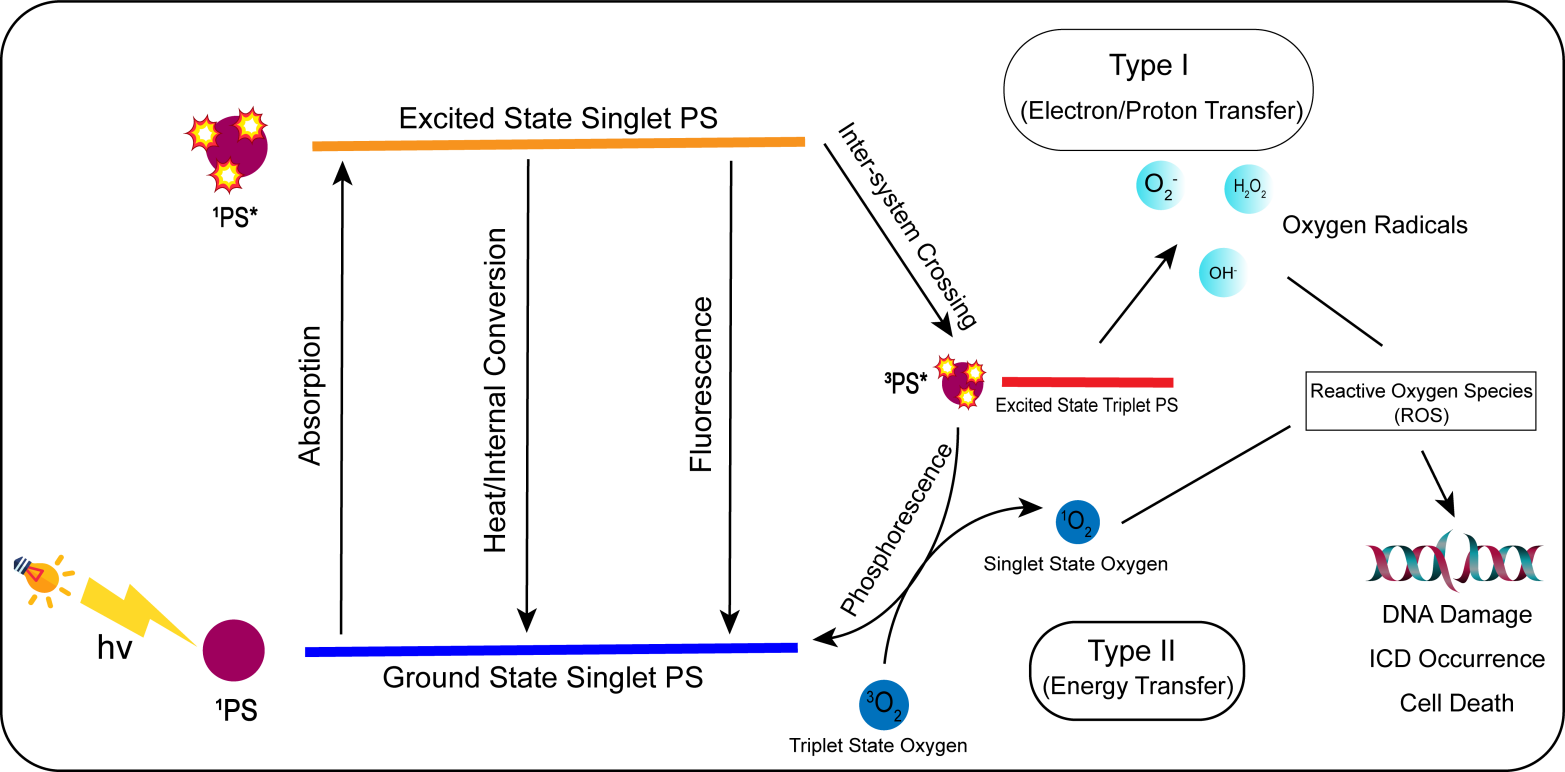
Mechanism of action of photodynamic therapy (PDT). In photodynamic treatment, the photosensitizer (PS) converts from a ground state to an excited singlet state by absorbing light energy when exposed to specific light wavelengths. The PS in the excited singlet state is unstable, and its energy is internally converted, lost as thermal energy, or radiated as fluorescence. The PS in the excited singlet state will reach the excited triplet state by inter-system crossing. The PS in the excited triplet state can be transformed into the ground singlet state through phosphorescence. In type I, the excited triplet state can generate oxygen radicals such as H_2_O_2_, OH^-^, and ${\text{O}}_{2}^{-}$ through electron/proton transfer. In type II, the excited triplet PS can convert ^3^O_2_ to singlet oxygen (^1^O_2_) by energy transfer. H_2_O_2_, OH-, O_2_-, and ^1^O_2_ are all reactive oxygen species, which can induce ICD reaction, DNA damage, and ultimately tumor cell death when acting on tumor cells.

PDT is a new type of tumor treatment that produces biological effects through photophysical and photochemical processes. In general, photosensitizers (PSs) are non-toxic photosensitizing dyes used during PDT. PSs selectively accumulate in tumor cells, and the sites of PS accumulation are irradiated with specific wavelengths of light. Furthermore, PSs can be activated *via* photophysical processes. In the presence of oxygen in cells and tissues, PSs produce cytotoxic substances and affect tumor cell signaling pathways, thus inducing tumor cell death and damaging tumor structures (203). The mechanism of action (123) can be divided into Type I and Type II reactions. Type I reactions produce oxidation products *via* electron transfer with cellular substrates, which then induce tumor cell death. Type II reactions transfer energy to produce highly reactive singlet oxygen, which is very powerful and can cause significant damage to tumor cells (**Figure 3**).

#### 5.1.1 Conventional photodynamic therapy

An increasing number of photosensitizers are being discovered and actively used in lung (204), prostate (205), head and neck (206), esophageal (207), and other cancers in clinical treatment. Porphyrins (PZ I/III) and 8-methoxy psoralen (8-MOP) are two typical photosensitizers that are non-toxic. When acting on tumor cells, they can reduce tumor cell viability, at the same time trigger ROS-related endoplasmic reticulum stress response, induce activation of the PERK signaling pathway, and phosphorylate EIF2α and ICD-related DAMPs, including CRT exposure and release of HMGB1 and type I interferon. The release of these DAMPs suggests that ICD response occurred in tumor cells after the administration of PDT. At the same time, cytotoxic T lymphocytes proliferated, DC activation and maturation occurred, co-stimulatory CD80 and 86 surface molecules were upregulated, immunosuppressive cells such as Tregs were reduced, and the TME was significantly improved and promoted the apoptosis of tumor cells (127, 190, 191).

#### 5.1.2 Application of near-infrared light immunotherapy

Near-infrared photoimmunotherapy (NIR-PIT) is a novel physical tumor therapy that combines photodynamic and targeted therapies. This treatment uses a targeted PS that recognizes and binds to specific monoclonal antibodies on the surface of tumor cells and induces cell death by exciting the PS with 690 nm NIR light. NIR light can penetrate several centimeters of tissues without harming DNA or normal cells, and the PS combines with specific proteins on the surface of tumor cells. The process of NIR light irradiation is only highly lethal to the target cells when combined with the PS and does not cause harm to normal cells, which significantly reduces the probability of side effects and improves the efficacy of tumor treatment (208, 209). This treatment method is currently used for urological (210), gastric (211), and head and neck cancers (212).

IRDye700DX is a targeted PS commonly used in NIR-PIT, which is water-soluble, non-toxic, and non-biotoxic. Moreover, it binds specifically to specific proteins on the surface of tumor cells, with unsuccessfully bound photosensitizers excreted in the urine (126).

In preclinical studies, NIR-PIT acts on skin squamous cell carcinoma cells, which swell and rupture, causing rapid and irreversible damage, during which the tumor cells undergo an ICD response, and CRT and HSP expression increases on the cell surface. Simultaneously, immunogenic signals such as ATP and HMGB1 are rapidly released, rapid DC activation and maturation occurs, and the anticancer immune response is enhanced. Furthermore, cytotoxic T lymphocytes proliferate in large numbers, immunosuppressive cells such as Tregs decrease in large numbers, tumor cells die in large numbers, and antitumor efficacy is significantly enhanced (192).

One study combined IRDye700DX with fibroblast activation protein-specific antibodies to target tumor-associated fibroblasts. After NIR-PIT acted on tumor-associated fibroblasts, esophageal tumor cells with radiotherapy resistance were re-sensitized to radiotherapy, and many tumor cells died after treatment compared to the number that died before NIR-PIT (193).

NIR-PIT is currently in phase 3 clinical trials for recurrent head and neck squamous cell carcinoma (213). Previous clinical trials have shown that NIR-PIT is well tolerated and that patient response and survival rates after treatment are positive and clinically meaningful for this disease (214).

In conclusion, conventional PDT or targeted therapy, mediated by NIR light, are noninvasive, highly spatially specific, and have low systemic toxicity. They can induce ICD response in tumor cells, eliminate tumor cell drug resistance, and promote tumor cell death. Their safe and effective anticancer effects provide a reliable alternative for patients with tumors suffering from underlying diseases who cannot tolerate chemotherapy (215).

### 5.2 Nano-pulse stimulation technology

Nano-pulse stimulation (NPS) is the application of ultrafast pulses of high irradiation energy to tumorigenic tissue, which alters the permeability of tumor cell membranes and produces a wide range of physiological responses (216, 217). The most significant changes were observed in Ca^2+^, where it was internally rearranged and translocated, and the endoplasmic reticulum stress response occurred under the effect of NPS, along with a significant release of ICD-related DAMPs such as heat shock proteins, ATP, and HMGB1, suggesting that NPS induced an ICD response in tumor cells. The effect of NPS resulted in a significant reduction of immunosuppressive cells such as Tregs, DC maturation and activation, massive death of tumor cells, enhanced antitumor immune effects, and inhibition of distant metastases. NPS is another application of nanotechnology, as a highly efficacious inducer of ICD, and its unique therapeutic advantages provide a new option for nanotechnological tumor treatment (194, 218, 219).

### 5.3 Carrier-mediated ICD induction

To further improve the precision of ICD inducers on specific tumor cells, the use of vectors combined with conventional ICD inducers on tumor cells has become a new research hotspot. Depending on their source, we classify these carriers as exogenous or endogenous. Exogenous carriers are substances artificially tailored with specific nanomaterials that can respond to specific stimuli, such as pH, temperature, and light, to release drugs at specific sites. The exogenous carriers include PLA-HES-FA (PHF) as well as HPMA copolymers. Unlike exogenous carriers, endogenous carriers are naturally present in cells or body fluids. Compared with exogenous carriers, endogenous carriers have natural advantages, including lower immunogenicity and biotoxicity as well as higher stability and delivery efficiency. Endogenous carriers include cell membrane carriers, liposomes, and exosomes (220, 221).

Using carrier-mediated ICD inducers to act on tumor cells can protect inducers from misidentification by the immune system and rapid removal. Simultaneously, vector-mediated ICD inducers can accumulate in tumor cells and increase the ICD response, further enhancing antitumor efficacy. Owing to the high precision of vector-mediated ICD inducers, adverse reactions during treatment were significantly reduced, and the survival rate of patients was improved during treatment (11, 222).

#### 5.3.1 Exogenous vector-mediated ICD induction

CPT-SS-NLG919 (CN) is an indoleamine 2,3-dioxygenase (IDO) inhibitor. High concentrations of reducing substances in tumor cells can degrade CN into camptothecin (CPT) and NLG919 (NLG), which can induce ICD, inhibit IDO activity, and improve the inhibitory TME. However, the disadvantage of these two substances is that they are poorly soluble and require high concentrations in tumor cells to be effective. One study combined the synthetic nanocarrier PHF with CN to form a CN@PHF. This carrier conjugate has a high drug-loading capacity and stability under neutral conditions, with good targeting properties, and can accumulate rapidly in tumor cells with a high concentration of CN. After CN@PHF acted on tumor cells, a high concentration of CN was degraded to CPT and NLG by the action of reducing substances. CPT induces ICD in tumor cells; many DAMPs associated with ICD are released, and many tumor cells die. At the same time, NLG in CN inhibits IDO activity and improves the immunosuppressive TME. During the immune response, DC cells mature and activate, immunosuppressive cells such as Tregs are significantly reduced, proinflammatory cytokines are released, cytotoxic T lymphocytes proliferate, and the tumor development process is hindered. Compared to the direct action of CN on tumor cells, the use of vectors to transport CN to tumor cells enhances the cytotoxic effect of the immune process, with a reduced probability of adverse effects and an increased survival time for the mouse. This therapeutic approach offers a new option for future tumor treatment (195).

HPMA copolymer is also a novel drug delivery carrier with good biocompatibility, water solubility, non-toxicity, and passive targeting, which can preferentially accumulate at tumor sites and reduce drug toxicity (223, 224). When adriamycin was encapsulated in the HPMA copolymer and acted on tumor cells, the expression of PI3K was significantly reduced in tumor cells under the action of nanoformulations, PI3K signaling pathway was inhibited, and tumor cells were sensitive to chemotherapeutic drugs. At the same time, a large amount of ROS was released, CRT was heavily expressed on the surface, ATP and HMGB1 were released in large amounts, and ICD response was further enhanced. Moreover, Tregs and other immunosuppressive cells were significantly reduced, proinflammatory cytokines were released, cytotoxic T lymphocytes proliferated, antitumor immune response was enhanced, and tumor cells died in large numbers (196, 197).

In conclusion, synthetic nanocarrier-mediated antitumor drug therapy can significantly improve the efficacy of tumor treatment in patients. However, synthetic nanocarriers also have some drawbacks, including unknown risks to patients due to the potential toxic effects of chemical drugs; therefore, there is a long way to go before the widespread use of synthetic nanocarriers in clinical tumor treatment (225).

#### 5.3.2 Endogenous vector-mediated ICD induction

**Figure 4:**
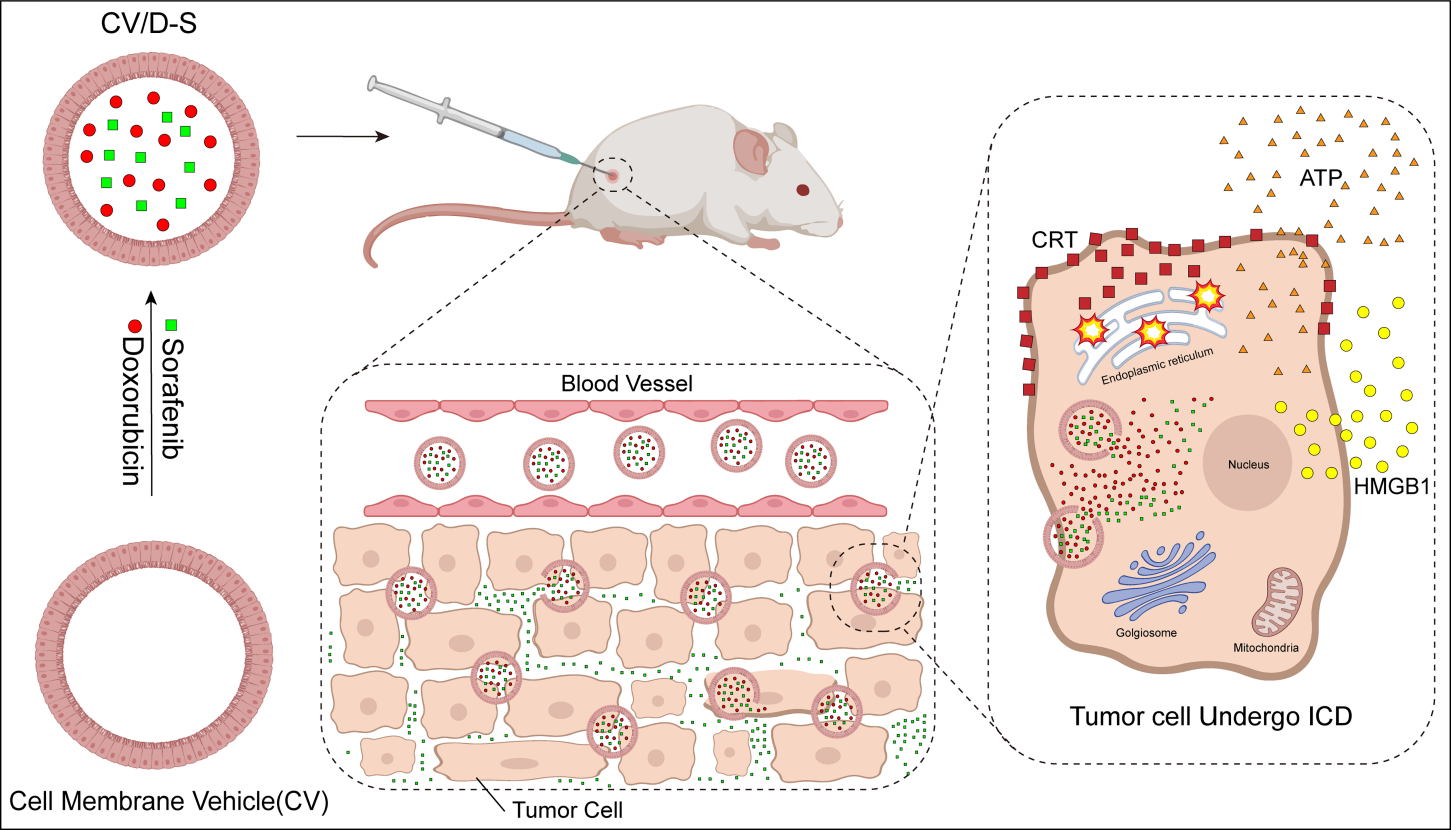
Mechanism of action of cell membrane vehicle induced immunogenic cell death (ICD) response. Doxorubicin (DOX) and sorafenib (SFN) were encapsulated in a cell membrane vehicle to form a highly stable spherical particle CV/D-S. After injecting CV/D-S into experimental animals, the particle flowed along the blood vessels to the location of tumor tissues and accumulated in large quantities at the site of tumorigenesis. The particle released SFN to regulate the TME. DOX induced ICD in tumor cells after entry.

Cell membrane vehicles (CVs) are derived from tumor cell membranes and are endogenous carriers with a structure similar to that of the parent body. They have the same tumor-targeting properties and are a novel drug delivery system (226). The ICD inducers doxorubicin (DOX) and sorafenib (SFN) were co-encapsulated in the CV; this vehicle conjugate is referred to as CV/D-S. Upon arrival at tumor cells, CV/D-S transports DOX to induce an ICD response in tumor cells, and the SFN drug is able to alter the TME in a dose-dependent manner. Compared with the application of antitumor drugs alone, the tumor cell ICD was further enhanced, DC maturation and activation occurred, cytotoxic T lymphocytes proliferated in large numbers, Treg immunosuppressive cells decreased in large numbers, and tumor cell apoptosis increased (**Figure 4**). CV/D-S is a spherical particle with good stability and high biocompatibility, and its use enables adequate drug loading and safe delivery of DOX and SFN drugs *in vivo*. Owing to the tumor homing properties of these CVs, CV/D-S can accumulate in large quantities at the site of tumorigenesis and exert excellent antitumor properties, making it a promising drug delivery platform that can improve the effectiveness of multiple tumor treatments (198, 199, 227).

Exosomes, another type of endogenous carriers, are vesicular structures actively secreted by cells. They are smaller, contain various bioactive substances, are less immunogenic, and can avoid misidentification and phagocytosis by the immune system (228, 229). One study encapsulated oxaliplatin ICD inducer in exosomes (IEXO-OXA). After IEXO-OXA acted on the tumor cells, the cytotoxicity of the chemotherapeutic drug increased, and the ICD response was further enhanced. Furthermore, the immunosuppressed TME was significantly improved, cytotoxic T lymphocytes were heavily activated, proinflammatory cytokines were also heavily increased, tumor cell activity was reduced, and apoptosis increased (200). In conclusion, as a targeted drug delivery system, exosomes can promote drug accumulation at the site of tumorigenesis, reduce the systemic distribution of antitumor drugs, and minimize side effects. Additionally, these endogenous carriers can be combined with a variety of therapeutic drugs, and their highly engineered characteristics can provide new options for various tumor treatments (201).

Carrier drug delivery systems can promote physical therapy of tumor cells by altering the cellular state. Liposomes are bilayer vesicular structures in cells that are capable of loading hydrophilic/hydrophobic drugs and delivering them into cells through endocytosis/fusion/activation of target cells by specific ligands. The liposome-mediated drug delivery system can maintain a particular drug concentration in the plasma for a long time, reduce the frequency of drug administration, and further improve bioavailability and safety (230, 231). To solve the problem of tumor cell hypoxia, hemoglobin and adriamycin are co-encapsulated in liposomes (DOX-Hb-Lipo), which have high oxygen-binding capacity and can effectively relieve tumor tissue hypoxia. In contrast, tumor cells produce a large amount of ROS, which mediate the ICD of tumor cells. CRT, HMGB1, and other ICD-related DAMP levels were significantly increased compared to those achieved with radiotherapy alone, tumor cell growth was inhibited, and the tumor-killing effect was enhanced considerably (202, 232).

#### 5.3.3 Advantages and limitations of carrier technology

In recent years, an increasing number of nanotechnology-based drug carrier systems have been developed, and both endogenous and synthetic carriers have been widely used in tumor therapy. The combined action of nanocarriers and ICD-inducing agent-based antitumor drugs has changed the immunosuppressive TME. Moreover, because of the precise targeting action of nanocarriers, the systemic distribution of antitumor drugs during administration is reduced, the amount of drug accumulation at specific locations and the duration of drug action are increased, the frequency of drug administration is reduced, and the side effects are significantly reduced. Antitumor immunotherapy is considerably enhanced and many tumor cells die. However, there are limitations in the development of nanotechnology, including the material, size, and concentration of nanoparticles, which may cause specific toxicity to cells, and artificial nanoparticles may be recognized as “foreign substances” by the body, and then erroneously engulfed and eliminated by the immune system. Most importantly, nanotechnology research is extremely difficult and requires increased funding, which hinders the full commercialization of nanotechnology and makes the universal clinical treatment using this technology difficult (233–235).

In conclusion, the clinical application of nanotechnology-based drug carrier systems has a positive and far-reaching impact on the clinical treatment of tumors. However, the limitations of its application must be addressed by further research.

## 6 Immunogenic cell death in clinical trials

With the widespread application of immunotherapy in clinical tumor treatment, research related to ICD inducers is also developing rapidly and has made specific achievements. Currently, many ICD induction methods have been applied in clinical trials as anticancer therapies to induce ICD responses in tumor cells, increase the apoptosis of tumor cells, and improve the prognosis of patients with tumors.

In a phase III clinical trial of metastatic colorectal cancer, FOLFOXIRI plus bevacizumab was administered to tumor cells. The chemical ICD-inducing oxaliplatin in the therapy enhanced DC function and induced ICD by increasing the exposure to tumor antigens. The trial results were positive, and this therapy has also been shown to be an option for the pretreatment of selected patients with metastatic colorectal cancer (236). Clinical trials using DC-based tumor vaccines with a chemical ICD-inducing agent for tumor cells are also underway. In phase I/II clinical trials of breast cancer, chemical ICD-inducing DOX and cyclophosphamide have been combined with a DC-based tumor vaccine to act on breast cancer cells. This resulted in an enhanced ICD response induced by the chemical ICD-inducing agent, a boost in the patient’s T-cell response to stimulation, and enhanced tumor immunogenicity. However, the effect of this immune response on patient survival is subject to ongoing observation (237). In addition, antigen-specific T cell activity in patients was enhanced in phase II clinical trials of ovarian cancer. This occurred after the DC-based tumor vaccine was administered to ovarian tumor cells with carboplatin and gemcitabine, which are chemical ICD inducers. The chemical ICD inducers in this therapy have been shown to function as active immune effector cells, and the tumor cell ICD response occurred together. This clinical trial in ovarian cancer prolonged survival of patients with ovarian tumors and gave strong confidence for further clinical trials of ICD (238).

The physical sources of ICD induction methods have also been used in clinical trials. Irreversible electroporation is a physical method that induces ICD response in tumor cells. In clinical trials in advanced pancreatic cancer, the combination of irreversible electroporation and Vγ9Vδ2 T cells acting on pancreatic tumor cells resulted in a decrease in tumor marker levels, enhanced antitumor immune effect, and a significant increase in survival of patients with advanced pancreatic cancer, compared to that before treatment (239).

Oncolytic viruses are typical pathogen-derived ICD inducers and positive findings have been achieved in clinical trials on oncolytic viruses. In phase I of a clinical study of recurrent malignant glioma, oncolytic viruses underwent an oncolytic response and induced an immune-related antitumor response after acting on glioma cells. The clinical trial results were promising, with 20% of the patients surviving more than 3 years after treatment. Furthermore, 60% of the patients who survived, experienced a 95% reduction in tumor volume, suggesting that oncolytic virus-induced antitumor immune responses may offer new hope for treating patients with recurrent malignant glioma (240). In addition, oncolytic viruses have been used in phase I clinical trials of multiple myeloma. After acting on myeloma cells, oncolytic viruses kill multiple myeloma cells while generating a solid and durable antitumor immune response in the patient’s body. When tumor-associated antigens are released into the periphery, cytotoxic T lymphocytes generate a strong antitumor immune response to the tumor-associated antigens, creating positive feedback until the effects caused by oncolytic viral infection are eliminated (241).

In conclusion, clinical trials related to ICD are progressing well. We believe that these ICD inducers can be used in clinical treatment and bring new hope to more patients with tumors.

## 7 Conclusion

In conclusion, owing to the low immunogenicity of tumor cells and tumor-killing immune cells, an immunosuppressive TME is formed during tumor treatment. The application of ICD inducers improves the immunogenicity of tumor cells and the TME. During ICD induction, CRT, ATP, HMGB1, and other DAMPs are released in large quantities, stimulating the activation of ICD-related signaling pathways, triggering the endoplasmic reticulum stress response, and promoting the onset of ICD response in tumor cells. ICD response promotes DC maturation/activation, increases the infiltration of cytotoxic T lymphocytes, and produces a more durable antitumor response.

ICD inducers play an important role in chemotherapy and physical therapy but are limited by toxicity and efficiency. The combined application of ICD inducers and nanotechnology-based ICD inducer delivery systems has emerged as a new technology that can substantially reduce the dosage and frequency of drugs used in tumor treatment through combined targeting, and can precisely achieve drug accumulation at the site of tumorigenesis. These new technologies have further improved the efficacy of antitumor treatment and reduced the occurrence of side effects, significantly improving the patient’s quality of life during the treatment process.

Whether using conventional methods or new technologies, ICD-inducing agents act on tumor cells through various mechanisms to induce the onset of the ICD response. However, most experiments are still in the primary research stage and have not been applied in clinical treatment. Additionally, the number and types of ICD inducers that have been elucidated and the tumor models used to verify the effects of these inducers are insufficient to meet the needs of human tumor treatment.

In the future, researchers need to discover more ICD inducers and develop new biomarkers and more diverse tumor models to further screen and validate the clinical effects of ICD inducers. Furthermore, clinical trials are being actively conducted to determine the exact mechanism of action, required dose, duration of treatment, and side effects of ICD inducers in the treatment of tumors to enable more types of ICD inducers to be used in clinical treatment in the future. In a word, ICD induction is a promising research field that requires further research to unravel the mysteries, and ICD response based on tumor cells will become the future trend in tumor treatment.

## Author contributions

GW and QW provided the article idea, DX conducted the initial research and participated in the writing. GW reviewed and revised the manuscript. All authors contributed to the manuscript and approved the submitted version.

## Funding

GW’s Liaoning Ph.D. Start-up Fund (2021-BS-209, Liaoning, 30,000CNY); GW’s Dalian Young Stars of Science and Technology (2021RQ010, Dalian, 100,000CNY); Liaoning Provincial Education Department (LJKZ0860).

## Conflict of interest

The authors declare that the research was conducted in the absence of any commercial or financial relationships that could be construed as a potential conflict of interest.

## Publisher’s note

All claims expressed in this article are solely those of the authors and do not necessarily represent those of their affiliated organizations, or those of the publisher, the editors and the reviewers. Any product that may be evaluated in this article, or claim that may be made by its manufacturer, is not guaranteed or endorsed by the publisher.

## References

[1] Cao M Li H Sun D Chen W. Cancer burden of major cancers in China: A need for sustainable actions. Cancer Commun (Lond) (2020) 40:205–10. doi: 10.1002/cac2.12025 PMC7667573 32359212

[2] anyi. Available at: http://gco.iarc.fr/today/home.

[3] Qiu H Cao S Xu R. Cancer incidence, mortality, and burden in China: a time-trend analysis and comparison with the united states and united kingdom based on the global epidemiological data released in 2020. Cancer Commun (Lond) (2021) 41:1037–48. doi: 10.1002/cac2.12197 PMC8504144 34288593

[4] Stone JB DeAngelis LM. Cancer-treatment-induced neurotoxicity–focus on newer treatments. Nat Rev Clin Oncol (2016) 13:92–105. doi: 10.1038/nrclinonc.2015.152 26391778 PMC4979320

[5] Miller M Hanna N. Advances in systemic therapy for non-small cell lung cancer. BMJ (2021) 375:n2363. doi: 10.1136/bmj.n2363 34753715

[6] Yang Y. Cancer immunotherapy: harnessing the immune system to battle cancer. J Clin Invest (2015) 125:3335–7. doi: 10.1172/JCI83871 PMC4588312 26325031

[7] Zhang Y Zhang Z. The history and advances in cancer immunotherapy: understanding the characteristics of tumor-infiltrating immune cells and their therapeutic implications. Cell Mol Immunol (2020) 17:807–21. doi: 10.1038/s41423-020-0488-6 PMC7395159 32612154

[8] Tang D Kang R Berghe TV Vandenabeele P Kroemer G. The molecular machinery of regulated cell death. Cell Res (2019) 29:347–64. doi: 10.1038/s41422-019-0164-5 PMC6796845 30948788

[9] Santagostino SF Assenmacher C-A Tarrant JC Adedeji AO Radaelli E. Mechanisms of regulated cell death: Current perspectives. Vet Pathol (2021) 58:596–623. doi: 10.1177/03009858211005537 34039100

[10] Woo Y Lee H-J Jung YM Jung Y-J. Regulated necrotic cell death in alternative tumor therapeutic strategies. Cells (2020) 9:e2709. doi: 10.3390/cells9122709 PMC7767016 33348858

[11] Zhou J Wang G Chen Y Wang H Hua Y Cai Z. Immunogenic cell death in cancer therapy: Present and emerging inducers. J Cell Mol Med (2019) 23:4854–65. doi: 10.1111/jcmm.14356 PMC6653385 31210425

[12] Wang Q Ju X Wang J Fan Y Ren M Zhang H. Immunogenic cell death in anticancer chemotherapy and its impact on clinical studies. Cancer Lett (2018) 438:17–23. doi: 10.1016/j.canlet.2018.08.028 30217563

[13] Ruan H Leibowitz BJ Zhang L Yu J. Immunogenic cell death in colon cancer prevention and therapy. Mol Carcinog (2020) 59:783–93. doi: 10.1002/mc.23183 PMC7593824 32215970

[14] Kroemer G Galluzzi L Kepp O Zitvogel L. Immunogenic cell death in cancer therapy. Annu Rev Immunol (2013) 31:51–72. doi: 10.1146/annurev-immunol-032712-100008 23157435

[15] Casares N Pequignot MO Tesniere A Ghiringhelli F Roux S Chaput N. Caspase-dependent immunogenicity of doxorubicin-induced tumor cell death. J Exp Med (2005) 202:1691–701. doi: 10.1084/jem.20050915 PMC2212968 16365148

[16] Galluzzi L Vitale I Warren S Adjemian S Agostinis P Martinez AB. Consensus guidelines for the definition, detection and interpretation of immunogenic cell death. J Immunother Cancer (2020) 8:e000337. doi: 10.1136/jitc-2019-000337 32209603 PMC7064135

[17] Arneth B. Tumor microenvironment. Med (Kaunas) (2019) 56:e15. doi: 10.3390/medicina56010015 PMC7023392 31906017

[18] Krysko DV Agostinis P Krysko O Garg AD Bachert C Lambrecht BN. Emerging role of damage-associated molecular patterns derived from mitochondria in inflammation. Trends Immunol (2011) 32:157–64. doi: 10.1016/j.it.2011.01.005 21334975

[19] Brenner C Galluzzi L Kepp O Kroemer G. Decoding cell death signals in liver inflammation. J Hepatol (2013) 59:583–94. doi: 10.1016/j.jhep.2013.03.033 23567086

[20] Obeid M Tesniere A Ghiringhelli F Fimia GM Apetoh L Perfettini J-L. Calreticulin exposure dictates the immunogenicity of cancer cell death. Nat Med (2007) 13:54–61. doi: 10.1038/nm1523 17187072

[21] Wang WA Groenendyk J Michalak M. Calreticulin signaling in health and disease. Int J Biochem Cell Biol (2012) 44:842–6. doi: 10.1016/j.biocel.2012.02.009 22373697

[22] Zamanian M Veerakumarasivam A Abdullah S Rosli R. Calreticulin and cancer. Pathol Oncol Res (2013) 19:149–54. doi: 10.1007/s12253-012-9600-2 23392843

[23] Panaretakis T Kepp O Brockmeier U Tesniere A Bjorklund A-C Chapman DC. Mechanisms of pre-apoptotic calreticulin exposure in immunogenic cell death. EMBO J (2009) 28:578–90. doi: 10.1038/emboj.2009.1 PMC2657583 19165151

[24] Zitvogel L Kepp O Senovilla L Menger L Chaput N Kroemer G. Immunogenic tumor cell death for optimal anticancer therapy: the calreticulin exposure pathway. Clin Cancer Res (2010) 16:3100–4. doi: 10.1158/1078-0432.CCR-09-2891 20421432

[25] Fucikova J Spisek R Kroemer G Galluzzi L. Calreticulin and cancer. Cell Res (2021) 31:5–16. doi: 10.1038/s41422-020-0383-9 32733014 PMC7853084

[26] Pawaria S Binder RJ. CD91-dependent programming of T-helper cell responses following heat shock protein immunization. Nat Commun (2011) 2:521. doi: 10.1038/ncomms1524 22045000 PMC3356570

[27] Schcolnik-Cabrera A Oldak B Juárez M Cruz-Rivera M Flisser A Mendlovic F. Calreticulin in phagocytosis and cancer: opposite roles in immune response outcomes. Apoptosis (2019) 24:245–55. doi: 10.1007/s10495-019-01532-0 30929105

[28] Chen G Ward MF Sama AE Wang H. Extracellular HMGB1 as a proinflammatory cytokine. J Interferon Cytokine Res (2004) 24:329–33. doi: 10.1089/107999004323142187 15212706

[29] Chen R Kang R Tang D. The mechanism of HMGB1 secretion and release. Exp Mol Med (2022) 54:91–102. doi: 10.1038/s12276-022-00736-w 35217834 PMC8894452

[30] Fucikova J Kepp O Kasikova L Petroni G Yamazaki T Liu P. Detection of immunogenic cell death and its relevance for cancer therapy. Cell Death Dis (2020) 11:1013. doi: 10.1038/s41419-020-03221-2 33243969 PMC7691519

[31] van Beijnum JR Buurman WA Griffioen AW. Convergence and amplification of toll-like receptor (TLR) and receptor for advanced glycation end products (RAGE) signaling pathways *via* high mobility group B1 (HMGB1). Angiogenesis (2008) 11:91–9. doi: 10.1007/s10456-008-9093-5 18264787

[32] Lotze MT DeMarco RA. Dealing with death: HMGB1 as a novel target for cancer therapy. Curr Opin Investig Drugs (2003) 4:1405–9. 14763124

[33] Behl T Sharma E Sehgal A Kaur I Kumar A Arora R. Expatiating the molecular approaches of HMGB1 in diabetes mellitus: Highlighting signalling pathways *via* RAGE and TLRs. Mol Biol Rep (2021) 48:1869–81. doi: 10.1007/s11033-020-06130-x 33479829

[34] Lan J Luo H Wu R Wang J Zhou B Zhang Y. Internalization of HMGB1 (High mobility group box 1) promotes angiogenesis in endothelial cells. Arterioscler Thromb Vasc Biol (2020) 40:2922–40. doi: 10.1161/ATVBAHA.120.315151 32998518

[35] Vultaggio-Poma V Sarti AC Di Virgilio F. Extracellular ATP: A feasible target for cancer therapy. Cells (2020) 9:e2496. doi: 10.3390/cells9112496 PMC7698494 33212982

[36] Li X He S Ma B. Autophagy and autophagy-related proteins in cancer. Mol Cancer (2020) 19:12. doi: 10.1186/s12943-020-1138-4 31969156 PMC6975070

[37] Di Virgilio F Sarti AC Falzoni S De Marchi E Adinolfi E. Extracellular ATP and P2 purinergic signalling in the tumour microenvironment. Nat Rev Cancer (2018) 18:601–18. doi: 10.1038/s41568-018-0037-0 30006588

[38] Kepp O Bezu L Yamazaki T Di Virgilio F Smyth MJ Kroemer G. ATP and cancer immunosurveillance. EMBO J (2021) 40:e108130. doi: 10.15252/embj.2021108130 34121201 PMC8246257

[39] Troitskaya OS Novak DD Richter VA Koval OA. Immunogenic cell death in cancer therapy. Acta Naturae (2022) 14:40–53. doi: 10.32607/actanaturae.11523 35441043 PMC9013441

[40] Elliott MR Chekeni FB Trampont PC Lazarowski ER Kadl A Walk SF. Nucleotides released by apoptotic cells act as a find-me signal to promote phagocytic clearance. Nature (2009) 461:282–6. doi: 10.1038/nature08296 PMC2851546 19741708

[41] Ghiringhelli F Apetoh L Tesniere A Aymeric L Ma Y Ortiz C. Activation of the NLRP3 inflammasome in dendritic cells induces IL-1beta-dependent adaptive immunity against tumors. Nat Med (2009) 15:1170–8. doi: 10.1038/nm.2028 19767732

[42] Swanson KV Deng M Ting JP-Y. The NLRP3 inflammasome: molecular activation and regulation to therapeutics. Nat Rev Immunol (2019) 19:477–89. doi: 10.1038/s41577-019-0165-0 PMC7807242 31036962

[43] Wu J Liu T Rios Z Mei Q Lin X Cao S. Heat shock proteins and cancer. Trends Pharmacol Sci (2017) 38:226–56. doi: 10.1016/j.tips.2016.11.009 28012700

[44] Yun CW Kim HJ Lim JH Lee SH. Heat shock proteins: Agents of cancer development and therapeutic targets in anti-cancer therapy. Cells (2019) 9:e60. doi: 10.3390/cells9010060 PMC7017199 31878360

[45] Zhu M Yang M Zhang J Yin Y Fan X Zhang Y. Immunogenic cell death induction by ionizing radiation. Front Immunol (2021) 12:705361. doi: 10.3389/fimmu.2021.705361 34489957 PMC8417736

[46] Kumar S Stokes J Singh UP Scissum Gunn K Acharya A Manne U. Targeting Hsp70: A possible therapy for cancer. Cancer Lett (2016) 374:156–66. doi: 10.1016/j.canlet.2016.01.056 PMC5553548 26898980

[47] Hoter A El-Sabban ME Naim HY. The HSP90 family: Structure, regulation, function, and implications in health and disease. Int J Mol Sci (2018) 19:e2560. doi: 10.3390/ijms19092560 PMC6164434 30158430

[48] Li J Buchner J. Structure, function and regulation of the hsp90 machinery. BioMed J (2013) 36:106–17. doi: 10.4103/2319-4170.113230 23806880

[49] Ashrafizadeh M Farhood B Eleojo Musa A Taeb S Najafi M. Damage-associated molecular patterns in tumor radiotherapy. Int Immunopharmacol (2020) 86:106761. doi: 10.1016/j.intimp.2020.106761 32629409

[50] Rapoport BL Anderson R. Realizing the clinical potential of immunogenic cell death in cancer chemotherapy and radiotherapy. Int J Mol Sci (2019) 20:e959. doi: 10.3390/ijms20040959 PMC6412296 30813267

[51] Zhang S-Y Boisson-Dupuis S Chapgier A Yang K Bustamante J Puel A. Inborn errors of interferon (IFN)-mediated immunity in humans: insights into the respective roles of IFN-alpha/beta, IFN-gamma, and IFN-lambda in host defense. Immunol Rev (2008) 226:29–40. doi: 10.1111/j.1600-065X.2008.00698.x 19161414

[52] Carrero JA. Confounding roles for type I interferons during bacterial and viral pathogenesis. Int Immunol (2013) 25:663–9. doi: 10.1093/intimm/dxt050 PMC3839063 24158954

[53] Rizza P Moretti F Belardelli F. Recent advances on the immunomodulatory effects of IFN-alpha: implications for cancer immunotherapy and autoimmunity. Autoimmunity (2010) 43:204–9. doi: 10.3109/08916930903510880 20187707

[54] Jorgovanovic D Song M Wang L Zhang Y. Roles of IFN-γ in tumor progression and regression: a review. biomark Res (2020) 8:49. doi: 10.1186/s40364-020-00228-x 33005420 PMC7526126

[55] Takeda Y Azuma M Funami K Shime H Matsumoto M Seya T. Type I interferon-independent dendritic cell priming and antitumor T cell activation induced by a mycoplasma fermentans lipopeptide. Front Immunol (2018) 9:496. doi: 10.3389/fimmu.2018.00496 29593736 PMC5861346

[56] Jh O Mj K Sj C Yh B Hk L Ec S. Sustained type I interferon reinforces NK cell-mediated cancer immunosurveillance during chronic virus infection. Cancer Immunol Res (2019) 7(4):584–99. doi: 10.1158/2326-6066.CIR-18-0403 30808680

[57] Müller E Christopoulos PF Halder S Lunde A Beraki K Speth M. Toll-like receptor ligands and interferon-γ synergize for induction of antitumor M1 macrophages. Front Immunol (2017) 8:1383. doi: 10.3389/fimmu.2017.01383 29123526 PMC5662546

[58] Gangaplara A Martens C Dahlstrom E Metidji A Gokhale AS Glass DD. Type I interferon signaling attenuates regulatory T cell function in viral infection and in the tumor microenvironment. PloS Pathog (2018) 14:e1006985. doi: 10.1371/journal.ppat.1006985 29672594 PMC5929570

[59] Garg AD Kaczmarek A Krysko O Vandenabeele P Krysko DV Agostinis P. ER stress-induced inflammation: does it aid or impede disease progression? Trends Mol Med (2012) 18:589–98. doi: 10.1016/j.molmed.2012.06.010 22883813

[60] Tesniere A Schlemmer F Boige V Kepp O Martins I Ghiringhelli F. Immunogenic death of colon cancer cells treated with oxaliplatin. Oncogene (2010) 29:482–91. doi: 10.1038/onc.2009.356 19881547

[61] Golden EB Frances D Pellicciotta I Demaria S Helen Barcellos-Hoff M Formenti SC. Radiation fosters dose-dependent and chemotherapy-induced immunogenic cell death. Oncoimmunology (2014) 3:e28518. doi: 10.4161/onci.28518 25071979 PMC4106151

[62] Li C Sun H Wei W Liu Q Wang Y Zhang Y. Mitoxantrone triggers immunogenic prostate cancer cell death *via* p53-dependent PERK expression. Cell Oncol (Dordr) (2020) 43:1099–116. doi: 10.1007/s13402-020-00544-2 PMC12990691 32710433

[63] Terenzi A Pirker C Keppler BK Berger W. Anticancer metal drugs and immunogenic cell death. J Inorg Biochem (2016) 165:71–9. doi: 10.1016/j.jinorgbio.2016.06.021 27350082

[64] Krysko DV Garg AD Kaczmarek A Krysko O Agostinis P Vandenabeele P. Immunogenic cell death and DAMPs in cancer therapy. Nat Rev Cancer (2012) 12:860–75 doi: 10.1038/nrc3380 23151605

[65] Xu D Wang Y Wu J Zhang Z Chen J Xie M. MTIF2 impairs 5 fluorouracil-mediated immunogenic cell death in hepatocellular carcinoma *in vivo*: Molecular mechanisms and therapeutic significance. Pharmacol Res (2021) 163:105265. doi: 10.1016/j.phrs.2020.105265 33129983

[66] Li X Zheng J Chen S Meng FD Ning J Sun SL. Oleandrin, a cardiac glycoside, induces immunogenic cell death *via* the PERK/elF2α/ATF4/CHOP pathway in breast cancer. Cell Death Dis (2021) 12:314. doi: 10.1038/s41419-021-03605-y 33762577 PMC7990929

[67] Sun F Cui L Li T Chen S Song J Li D. Oxaliplatin induces immunogenic cells death and enhances therapeutic efficacy of checkpoint inhibitor in a model of murine lung carcinoma. J Recept Signal Transduct Res (2019) 39:208–14. doi: 10.1080/10799893.2019.1655050 31441696

[68] Zhu H Shan Y Ge K Lu J Kong W Jia C. Oxaliplatin induces immunogenic cell death in hepatocellular carcinoma cells and synergizes with immune checkpoint blockade therapy. Cell Oncol (Dordr) (2020) 43:1203–14. doi: 10.1007/s13402-020-00552-2 PMC12990673 32797385

[69] Wei W Li H Zhang G Zhang Y Wu K Bao R. Proteasome inhibitors attenuates mitoxantrone-triggered immunogenic cell death in prostate cancer cells. Med Oncol (2020) 37:116. doi: 10.1007/s12032-020-01445-y 33215275

[70] Lau TS Chan LKY Man GCW Wong CH Lee JHS Yim SF. Paclitaxel induces immunogenic cell death in ovarian cancer *via* TLR4/IKK2/SNARE-dependent exocytosis. Cancer Immunol Res (2020) 8:1099–111. doi: 10.1158/2326-6066.CIR-19-0616 32354736

[71] Qin T Xu X Zhang Z Li J You X Guo H. Paclitaxel/sunitinib-loaded micelles promote an antitumor response *in vitro* through synergistic immunogenic cell death for triple-negative breast cancer. Nanotechnology (2020) 31:365101. doi: 10.1088/1361-6528/ab94dc 32434167

[72] Nishimura J Deguchi S Tanaka H Yamakoshi Y Yoshii M Tamura T. Induction of immunogenic cell death of esophageal squamous cell carcinoma by 5-fluorouracil and cisplatin. In Vivo (2021) 35:743–52. doi: 10.21873/invivo.12315 PMC8045060 33622867

[73] Li Y Zhang H Li Q Zou P Huang X Wu C. CDK12/13 inhibition induces immunogenic cell death and enhances anti-PD-1 anticancer activity in breast cancer. Cancer Lett (2020) 495:12–21. doi: 10.1016/j.canlet.2020.09.011 32941949

[74] Lotsberg ML Wnuk-Lipinska K Terry S Tan TZ Lu N Trachsel-Moncho L. AXL targeting abrogates autophagic flux and induces immunogenic cell death in drug-resistant cancer cells. J Thorac Oncol (2020) 15:973–99. doi: 10.1016/j.jtho.2020.01.015 PMC7397559 32018052

[75] Petrazzuolo A Perez-Lanzon M Liu P Maiuri MC Kroemer G. Crizotinib and ceritinib trigger immunogenic cell death *via* on-target effects. Oncoimmunology (2021) 10:1973197. doi: 10.1080/2162402X.2021.1973197 34712511 PMC8547833

[76] Zhou J Yang Q Lu L Tuo Z Shou Z Cheng J. PLK1 inhibition induces immunogenic cell death and enhances immunity against NSCLC. Int J Med Sci (2021) 18:3516–25. doi: 10.7150/ijms.60135 PMC8436107 34522178

[77] Huang Z Hu H. Arginine deiminase induces immunogenic cell death and is enhanced by n-acetylcysteine in murine MC38 colorectal cancer cells and MDA-MB-231 human breast cancer cells *In vitro* . Molecules (2021) 26:511. doi: 10.3390/molecules26020511 33478072 PMC7835909

[78] Tong J Tan X Risnik D Gao M Song X Ermine K. BET protein degradation triggers DR5-mediated immunogenic cell death to suppress colorectal cancer and potentiate immune checkpoint blockade. Oncogene (2021) 40:6566–78. doi: 10.1038/s41388-021-02041-8 PMC8642302 34615996

[79] Bi F Jiang Z Park W Hartwich TMP Ge Z Chong KY. A benzenesulfonamide-based mitochondrial uncoupler induces endoplasmic reticulum stress and immunogenic cell death in epithelial ovarian cancer. Mol Cancer Ther (2021) 20:2398–409. doi: 10.1158/1535-7163.MCT-21-0396 PMC8643344 34625503

[80] Montes de Oca R Alavi AS Vitali N Bhattacharya S Blackwell C Patel K. Belantamab mafodotin (GSK2857916) drives immunogenic cell death and immune-mediated antitumor responses *In vivo* . Mol Cancer Ther (2021) 20:1941–55. doi: 10.1158/1535-7163.MCT-21-0035 PMC9398105 34253590

[81] Wang L Guan R Xie L Liao X Xiong K Rees TW. An ER-targeting Iridium(III) complex that induces immunogenic cell death in non-Small-Cell lung cancer. Angew Chem Int Ed Engl (2021) 60:4657–65. doi: 10.1002/anie.202013987 33217194

[82] Wernitznig D Kiakos K Del Favero G Harrer N Machat H Osswald A. First-in-class ruthenium anticancer drug (KP1339/IT-139) induces an immunogenic cell death signature in colorectal spheroids *in vitro* . Metallomics (2019) 11:1044–8. doi: 10.1039/c9mt00051h 30942231

[83] Wernitznig D Meier-Menches SM Cseh K Theiner S Wenisch D Schweikert A. Plecstatin-1 induces an immunogenic cell death signature in colorectal tumour spheroids. Metallomics (2020) 12:2121–33. doi: 10.1039/d0mt00227e 33295928

[84] Kaur P Johnson A Northcote-Smith J Lu C Suntharalingam K. Immunogenic cell death of breast cancer stem cells induced by an endoplasmic reticulum-targeting Copper(II) complex. Chembiochem (2020) 21:3618–24. doi: 10.1002/cbic.202000553 PMC7757018 32776422

[85] You SY Rui W Chen ST Chen HC Liu XW Huang J. Process of immunogenic cell death caused by disulfiram as the anti-colorectal cancer candidate. Biochem Biophys Res Commun (2019) 513:891–7. doi: 10.1016/j.bbrc.2019.03.192 31003768

[86] Fletcher R Tong J Risnik D Leibowitz BJ Wang Y-J Concha-Benavente F. Non-steroidal anti-inflammatory drugs induce immunogenic cell death in suppressing colorectal tumorigenesis. Oncogene (2021) 40:2035–50. doi: 10.1038/s41388-021-01687-8 PMC7981263 33603166

[87] Uscanga-Palomeque AC Calvillo-Rodríguez KM Gómez-Morales L Lardé E Denèfle T Caballero-Hernández D. CD47 agonist peptide PKHB1 induces immunogenic cell death in T-cell acute lymphoblastic leukemia cells. Cancer Sci (2019) 110:256–68. doi: 10.1111/cas.13885 PMC6317946 30460757

[88] Calvillo-Rodríguez KM Mendoza-Reveles R Gómez-Morales L Uscanga-Palomeque AC Karoyan P Martínez-Torres AC. PKHB1, a thrombospondin-1 peptide mimic, induces anti-tumor effect through immunogenic cell death induction in breast cancer cells. Oncoimmunology (2022) 11:2054305. doi: 10.1080/2162402X.2022.2054305 35402082 PMC8986196

[89] Zhu C Ren X Liu D Zhang C. Oxaliplatin-induced hepatic sinusoidal obstruction syndrome. Toxicology (2021) 460:152882. doi: 10.1016/j.tox.2021.152882 34352347

[90] Yamazaki T Buqué A Ames TD Galluzzi L. PT-112 induces immunogenic cell death and synergizes with immune checkpoint blockers in mouse tumor models. Oncoimmunology (2020) 9:1721810. doi: 10.1080/2162402X.2020.1721810 32117585 PMC7028345

[91] Karp DD Camidge DR Infante JR Ames TD Price MR Jimeno J. Phase I study of PT-112, a novel pyrophosphate-platinum immunogenic cell death inducer, in advanced solid tumours. EClinicalMedicine (2022) 49:101430. doi: 10.1016/j.eclinm.2022.101430 35747193 PMC9156977

[92] Karp DD Camidge DR Infante JR Ames TD Jimeno JM Bryce AH. PT-112: A well-tolerated novel immunogenic cell death (ICD) inducer with activity in advanced solid tumors. Ann Oncol (2018) 29:viii143. doi: 10.1093/annonc/mdy279.424

[93] Martins-Teixeira MB Carvalho I. Antitumour anthracyclines: Progress and perspectives. ChemMedChem (2020) 15:933–48. doi: 10.1002/cmdc.202000131 32314528

[94] Mitoxantrone. LiverTox: Clinical and research information on drug-induced liver injury (2012). Bethesda (MD: National Institute of Diabetes and Digestive and Kidney Diseases. Available at: http://www.ncbi.nlm.nih.gov/books/NBK547931/ (Accessed July 20, 2022). 31643176

[95] Gallego-Jara J Lozano-Terol G Sola-Martínez RA Cánovas-Díaz M de Diego Puente T. A compressive review about taxol®: History and future challenges. Molecules (2020) 25:e5986. doi: 10.3390/molecules25245986 PMC7767101 33348838

[96] Kumavath R Paul S Pavithran H Paul MK Ghosh P Barh D. Emergence of cardiac glycosides as potential drugs: Current and future scope for cancer therapeutics. Biomolecules (2021) 11:1275. doi: 10.3390/biom11091275 34572488 PMC8465509

[97] Reddy D Kumavath R Ghosh P Barh D. Lanatoside c induces G2/M cell cycle arrest and suppresses cancer cell growth by attenuating MAPK, wnt, JAK-STAT, and PI3K/AKT/mTOR signaling pathways. Biomolecules (2019) 9:e792. doi: 10.3390/biom9120792 PMC6995510 31783627

[98] Kaushik V Yakisich JS Azad N Kulkarni Y Venkatadri R Wright C. Anti-tumor effects of cardiac glycosides on human lung cancer cells and lung tumorspheres. J Cell Physiol (2017) 232:2497–507. doi: 10.1002/jcp.25611 27662422

[99] Felth J Rickardson L Rosén J Wickström M Fryknäs M Lindskog M. Cytotoxic effects of cardiac glycosides in colon cancer cells, alone and in combination with standard chemotherapeutic drugs. J Nat Prod (2009) 72:1969–74. doi: 10.1021/np900210m 19894733

[100] Elmaci İ Alturfan EE Cengiz S Ozpinar A Altinoz MA. Neuroprotective and tumoricidal activities of cardiac glycosides. could oleandrin be a new weapon against stroke and glioblastoma? Int J Neurosci (2018) 128:865–77. doi: 10.1080/00207454.2018.1435540 29383986

[101] Zhang C Xie S-H Xu B Lu S Liu P. Digitalis use and the risk of breast cancer: A systematic review and meta-analysis. Drug Saf (2017) 40:285–92. doi: 10.1007/s40264-016-0484-z 28130772

[102] Roskoski R. Properties of FDA-approved small molecule protein kinase inhibitors: A 2022 update. Pharmacol Res (2022) 175:106037. doi: 10.1016/j.phrs.2021.106037 34921994

[103] Roskoski R. Properties of FDA-approved small molecule protein kinase inhibitors: A 2021 update. Pharmacol Res (2021) 165:105463. doi: 10.1016/j.phrs.2021.105463 33513356

[104] Roskoski R Jr. Properties of FDA-approved small molecule protein kinase inhibitors: A 2020 update. Pharmacol Res (2020) 152:104609. doi: 10.1016/j.phrs.2019.104609 31862477

[105] Scirocchi F Napoletano C Pace A Rahimi Koshkaki H Di Filippo A Zizzari IG. Immunogenic cell death and immunomodulatory effects of cabozantinib. Front Oncol (2021) 11:755433. doi: 10.3389/fonc.2021.755433 34745989 PMC8564482

[106] Junwei S Vakoc CR. The mechanisms behind the therapeutic activity of BET bromodomain inhibition. Mol Cell (2014) 54:728–36. doi: 10.1016/j.molcel.2014.05.016 PMC4236231 24905006

[107] Phillips MM Sheaff MT Szlosarek PW. Targeting arginine-dependent cancers with arginine-degrading enzymes: Opportunities and challenges. Cancer Res Treat (2013) 45:251–62. doi: 10.4143/crt.2013.45.4.251 PMC3893322 24453997

[108] Patil M Bhaumik J Babykutty S Banerjee U Fukumura D. Arginine dependence of tumor cells: targeting a chink in cancer’s armor. Oncogene (2016) 35:4957–72. doi: 10.1038/onc.2016.37 PMC5457742 27109103

[109] Delmore JE Issa GC Lemieux ME Rahl PB Shi J Jacobs HM. BET bromodomain inhibition as a therapeutic strategy to target c-myc. Cell (2011) 146:904–17. doi: 10.1016/j.cell.2011.08.017 PMC3187920 21889194

[110] DeBerardinis RJ Chandel NS. We need to talk about the warburg effect. Nat Metab (2020) 2:127–9. doi: 10.1038/s42255-020-0172-2 32694689

[111] Chuang C-H Dorsch M Dujardin P Silas S Ueffing K Hölken JM. Altered mitochondria functionality defines a metastatic cell state in lung cancer and creates an exploitable vulnerability. Cancer Res (2021) 81:567–79. doi: 10.1158/0008-5472.CAN-20-1865 PMC8137518 33239425

[112] Vasan K Werner M Chandel NS. Mitochondrial metabolism as a target for cancer therapy. Cell Metab (2020) 32:341–52. doi: 10.1016/j.cmet.2020.06.019 PMC7483781 32668195

[113] Childress ES Alexopoulos SJ Hoehn KL Santos WL. Small molecule mitochondrial uncouplers and their therapeutic potential. J Med Chem (2018) 61:4641–55. doi: 10.1021/acs.jmedchem.7b01182 29156129

[114] Ss Pindiprolu SK Krishnamurthy PT Ghanta VR Chintamaneni PK. Phenyl boronic acid-modified lipid nanocarriers of niclosamide for targeting triple-negative breast cancer. Nanomed (Lond) (2020) 15:1551–65. doi: 10.2217/nnm-2020-0003 32618501

[115] Senkowski W Zhang X Olofsson MH Isacson R Höglund U Gustafsson M. Three-dimensional cell culture-based screening identifies the anthelmintic drug nitazoxanide as a candidate for treatment of colorectal cancer. Mol Cancer Ther (2015) 14:1504–16. doi: 10.1158/1535-7163.MCT-14-0792 25911689

[116] Alasadi A Chen M Swapna GVT Tao H Guo J Collantes J. Effect of mitochondrial uncouplers niclosamide ethanolamine (NEN) and oxyclozanide on hepatic metastasis of colon cancer. Cell Death Dis (2018) 9:215. doi: 10.1038/s41419-017-0092-6 29440715 PMC5833462

[117] Shrestha R Johnson E Byrne FL. Exploring the therapeutic potential of mitochondrial uncouplers in cancer. Mol Metab (2021) 51:101222. doi: 10.1016/j.molmet.2021.101222 33781939 PMC8129951

[118] Zhang Y Yang S Yang Y Liu T. Resveratrol induces immunogenic cell death of human and murine ovarian carcinoma cells. Infect Agent Cancer (2019) 14:27. doi: 10.1186/s13027-019-0247-4 31636696 PMC6798484

[119] Nawaz W Zhou Z Deng S Ma X Ma X Li C. Therapeutic versatility of resveratrol derivatives. Nutrients (2017) 9:1188. doi: 10.3390/nu9111188 29109374 PMC5707660

[120] Bacchi S Palumbo P Sponta A Coppolino MF. Clinical pharmacology of non-steroidal anti-inflammatory drugs: a review. Antiinflamm Antiallergy Agents Med Chem (2012) 11:52–64. doi: 10.2174/187152312803476255 22934743

[121] Zappavigna S Cossu AM Grimaldi A Bocchetti M Ferraro GA Nicoletti GF. Anti-inflammatory drugs as anticancer agents. Int J Mol Sci (2020) 21:e2605. doi: 10.3390/ijms21072605 PMC7177823 32283655

[122] Kozuki Y Miura Y Yagasaki K. Resveratrol suppresses hepatoma cell invasion independently of its anti-proliferative action. Cancer Lett (2001) 167:151–6. doi: 10.1016/s0304-3835(01)00476-1 11369135

[123] Chilakamarthi U Giribabu L. Photodynamic therapy: Past, present and future. Chem Rec (2017) 17:775–802. doi: 10.1002/tcr.201600121 28042681

[124] Fucikova J Moserova I Truxova I Hermanova I Vancurova I Partlova S. High hydrostatic pressure induces immunogenic cell death in human tumor cells. Int J Cancer (2014) 135:1165–77. doi: 10.1002/ijc.28766 24500981

[125] Yoon Y Ku B Lee K Jung YJ Baek SJ. Cold atmospheric plasma induces HMGB1 expression in cancer cells. Anticancer Res (2019) 39:2405–13. doi: 10.21873/anticanres.13358 31092433

[126] Kobayashi H Choyke PL. Near-infrared photoimmunotherapy of cancer. Acc Chem Res (2019) 52:2332–9. doi: 10.1021/acs.accounts.9b00273 PMC6704485 31335117

[127] Li W Yang J Luo L Jiang M Qin B Yin H. Targeting photodynamic and photothermal therapy to the endoplasmic reticulum enhances immunogenic cancer cell death. Nat Commun (2019) 10:3349. doi: 10.1038/s41467-019-11269-8 31350406 PMC6659660

[128] Podolska MJ Shan X Janko C Boukherroub R Gaipl US Szunerits S. Graphene-induced hyperthermia (GIHT) combined with radiotherapy fosters immunogenic cell death. Front Oncol (2021) 11:664615. doi: 10.3389/fonc.2021.664615 34485114 PMC8415397

[129] Voloshin T Kaynan N Davidi S Porat Y Shteingauz A Schneiderman RS. Tumor-treating fields (TTFields) induce immunogenic cell death resulting in enhanced antitumor efficacy when combined with anti-PD-1 therapy. Cancer Immunol Immunother (2020) 69:1191–204. doi: 10.1007/s00262-020-02534-7 PMC7303058 32144446

[130] Aryankalayil MJ Makinde AY Gameiro SR Hodge JW Rivera-Solis PP Palayoor ST. Defining molecular signature of pro-immunogenic radiotherapy targets in human prostate cancer cells. Radiat Res (2014) 182:139–48. doi: 10.1667/RR13731.1 PMC4216662 25003313

[131] Moserova I Truxova I Garg AD Tomala J Agostinis P Cartron PF. Caspase-2 and oxidative stress underlie the immunogenic potential of high hydrostatic pressure-induced cancer cell death. Oncoimmunology (2017) 6:e1258505. doi: 10.1080/2162402X.2016.1258505 28197379 PMC5283635

[132] Troitskaya O Golubitskaya E Biryukov M Varlamov M Gugin P Milakhina E. Non-thermal plasma application in tumor-bearing mice induces increase of serum HMGB1. Int J Mol Sci (2020) 21:e5128. doi: 10.3390/ijms21145128 PMC7404183 32698492

[133] Chandra RA Keane FK Voncken FEM Thomas CR. Contemporary radiotherapy: present and future. Lancet (2021) 398:171–84. doi: 10.1016/S0140-6736(21)00233-6 34166607

[134] Kamran SC D’amico AV. Radiation therapy for prostate cancer. Hematol/Oncol Clin North Am (2020) 34(1):45–69. doi: 10.1016/j.hoc.2019.08.017 31739952

[135] Shah C. Intraoperative radiation therapy for breast cancer: Are we there yet? Ann Surg Oncol (2021) 28:20–1. doi: 10.1245/s10434-020-09356-y 33206264

[136] Williamson CW Liu HC Mayadev J Mell LK. Advances in external beam radiation therapy and brachytherapy for cervical cancer. Clin Oncol (R Coll Radiol) (2021) 33:567–78. doi: 10.1016/j.clon.2021.06.012 34266728

[137] Pasquier D Lacornerie T Mirabel X Brassart C Vanquin L Lartigau E. Stereotactic body radiotherapy. how to better protect normal tissues? Cancer Radiother (2019) 23:630–5. doi: 10.1016/j.canrad.2019.07.153 31447339

[138] Vallard A Vial N Jmour O Rehailia-Blanchard A Trone J-C Sotton S. Stereotactic body radiotherapy: Passing fad or revolution? Bull Cancer (2020) 107:244–53. doi: 10.1016/j.bulcan.2019.09.011 31864665

[139] Singh AK Winslow TB Kermany MH Goritz V Heit L Miller A. A pilot study of stereotactic body radiation therapy combined with cytoreductive nephrectomy for metastatic renal cell carcinoma. Clin Cancer Res (2017) 23:5055–65. doi: 10.1158/1078-0432.CCR-16-2946 PMC5581708 28630212

[140] Adkins I Hradilova N Palata O Sadilkova L Palova-Jelinkova L Spisek R. High hydrostatic pressure in cancer immunotherapy and biomedicine. Biotechnol Adv (2018) 36:577–82. doi: 10.1016/j.biotechadv.2018.01.015 29409785

[141] Yan S Liu K Mu L Liu J Tang W Liu B. Research and application of hydrostatic high pressure in tumor vaccines (Review). Oncol Rep (2021) 45:75. doi: 10.3892/or.2021.8026 33760193 PMC8020208

[142] Rosenblatt J Avigan D. Cellular immunotherapy for multiple myeloma. Best Pract Res Clin Haematol (2008) 21:559–77. doi: 10.1016/j.beha.2008.07.007 18790455

[143] Jocham D Richter A Hoffmann L Iwig K Fahlenkamp D Zakrzewski G. Adjuvant autologous renal tumour cell vaccine and risk of tumour progression in patients with renal-cell carcinoma after radical nephrectomy: phase III, randomised controlled trial. Lancet (2004) 363:594–9. doi: 10.1016/S0140-6736(04)15590-6 14987883

[144] Adkins I Fucikova J Garg AD Agostinis P Špíšek R. Physical modalities inducing immunogenic tumor cell death for cancer immunotherapy. Oncoimmunology (2015) 3:e968434. doi: 10.4161/21624011.2014.968434 25964865 PMC4352954

[145] Lassche G Crezee J Van Herpen CML. Whole-body hyperthermia in combination with systemic therapy in advanced solid malignancies. Crit Rev Oncol Hematol (2019) 139:67–74. doi: 10.1016/j.critrevonc.2019.04.023 31112883

[146] Yan D Sherman JH Keidar M. Cold atmospheric plasma, a novel promising anti-cancer treatment modality. Oncotarget (2017) 8:15977–95. doi: 10.18632/oncotarget.13304 PMC5362540 27845910

[147] Motaln H Recek N Rogelj B. Intracellular responses triggered by cold atmospheric plasma and plasma-activated media in cancer cells. Molecules (2021) 26:1336. doi: 10.3390/molecules26051336 33801451 PMC7958621

[148] Tavares-da-Silva E Pereira E Pires AS Neves AR Braz-Guilherme C Marques IA. Cold atmospheric plasma, a novel approach against bladder cancer, with higher sensitivity for the high-grade cell line. Biol (Basel) (2021) 10:41. doi: 10.3390/biology10010041 PMC7828061 33435434

[149] Ha J-H Kim Y-J. Photodynamic and cold atmospheric plasma combination therapy using polymeric nanoparticles for the synergistic treatment of cervical cancer. Int J Mol Sci (2021) 22:1172. doi: 10.3390/ijms22031172 33504007 PMC7865232

[150] Estarabadi H Atyabi SA Tavakkoli S Noormohammadi Z Gholami MR Ghiaseddin A. Cold atmospheric plasma induced genotoxicity and cytotoxicity in esophageal cancer cells. Mol Biol Rep (2021) 48:1323–33. doi: 10.1007/s11033-021-06178-3 33547994

[151] Hua D Cai D Ning M Yu L Zhang Z Han P. Cold atmospheric plasma selectively induces G0/G1 cell cycle arrest and apoptosis in AR-independent prostate cancer cells. J Cancer (2021) 12:5977–86. doi: 10.7150/jca.54528 PMC8408125 34476012

[152] Mun EJ Babiker HM Weinberg U Kirson ED Von Hoff DD. Tumor-treating fields: A fourth modality in cancer treatment. Clin Cancer Res (2018) 24:266–75. doi: 10.1158/1078-0432.CCR-17-1117 28765323

[153] Voloshin T Schneiderman RS Volodin A Shamir RR Kaynan N Zeevi E. Tumor treating fields (TTFields) hinder cancer cell motility through regulation of microtubule and acting dynamics. Cancers (Basel) (2020) 12:E3016. doi: 10.3390/cancers12103016 PMC7603026 33080774

[154] Rominiyi O Vanderlinden A Clenton SJ Bridgewater C Al-Tamimi Y Collis SJ. Tumour treating fields therapy for glioblastoma: current advances and future directions. Br J Cancer (2021) 124:697–709. doi: 10.1038/s41416-020-01136-5 33144698 PMC7884384

[155] Davies AM Weinberg U Palti Y. Tumor treating fields: a new frontier in cancer therapy. Ann New York Acad Sci (2013) 1291:86–95. doi: 10.1111/nyas.12112 23659608

[156] Arvind R Chandana SR Borad MJ Pennington D Mody K Babiker H. Tumor-treating fields: A fourth modality in cancer treatment, new practice updates. Crit Rev Oncol/Hematol (2021) 168:103535. doi: 10.1016/j.critrevonc.2021.103535 34808377

[157] Rivera F Benavides M Gallego J Guillen-Ponce C Lopez-Martin J Küng M. Tumor treating fields in combination with gemcitabine or gemcitabine plus nab-paclitaxel in pancreatic cancer: Results of the PANOVA phase 2 study. Pancreatology (2019) 19:64–72. doi: 10.1016/j.pan.2018.10.004 30396819

[158] Vergote I von Moos R Manso L Van Nieuwenhuysen E Concin N Sessa C. Tumor treating fields in combination with paclitaxel in recurrent ovarian carcinoma: Results of the INNOVATE pilot study. Gynecol Oncol (2018) 150:471–7. doi: 10.1016/j.ygyno.2018.07.018 30060963

[159] Mehta M Gondi V Ahluwalia M Brown P. 1594TiP - radiosurgery followed by tumour treating fields (TTFields) for brain metastases (1-10) from NSCLC in the phase III METIS trial. Ann Oncol (2019) 30:v659. doi: 10.1093/annonc/mdz260.116

[160] Michl P Gress TM. Bacteria and bacterial toxins as therapeutic agents for solid tumors. Curr Cancer Drug Targets (2004) 4:689–702. doi: 10.2174/1568009043332727 15578923

[161] Sedighi M Zahedi Bialvaei A Hamblin MR Ohadi E Asadi A Halajzadeh M. Therapeutic bacteria to combat cancer; current advances, challenges, and opportunities. Cancer Med (2019) 8:3167–81. doi: 10.1002/cam4.2148 PMC6558487 30950210

[162] Tsun A Miao XN Wang CM Yu DC. Oncolytic immunotherapy for treatment of cancer. Adv Exp Med Biol (2016) 909:241–83. doi: 10.1007/978-94-017-7555-7_5 27240460

[163] Li C Zhang Y Yan S Zhang G Wei W Qi Z. Alternol triggers immunogenic cell death *via* reactive oxygen species generation. Oncoimmunology (2021) 10:1952539. doi: 10.1080/2162402X.2021.1952539 34350063 PMC8296969

[164] Oresta B Pozzi C Braga D Hurle R Lazzeri M Colombo P. Mitochondrial metabolic reprogramming controls the induction of immunogenic cell death and efficacy of chemotherapy in bladder cancer. Sci Transl Med (2021) 13:eaba6110. doi: 10.1126/scitranslmed.aba6110 33408185

[165] Ye T Jiang K Wei L Barr MP Xu Q Zhang G. Oncolytic Newcastle disease virus induces autophagy-dependent immunogenic cell death in lung cancer cells. Am J Cancer Res (2018) 8:1514–27. PMC6129498 30210920

[166] Ma J Ramachandran M Jin C Quijano-Rubio C Martikainen M Yu D. Characterization of virus-mediated immunogenic cancer cell death and the consequences for oncolytic virus-based immunotherapy of cancer. Cell Death Dis (2020) 11:48. doi: 10.1038/s41419-020-2236-3 31969562 PMC6976683

[167] Wang X Shao X Gu L Jiang K Wang S Chen J. Targeting STAT3 enhances NDV-induced immunogenic cell death in prostate cancer cells. J Cell Mol Med (2020) 24:4286–97. doi: 10.1111/jcmm.15089 PMC7171322 32100392

[168] Goradel NH Alizadeh A Hosseinzadeh S Taghipour M Ghesmati Z Arashkia A. Oncolytic virotherapy as promising immunotherapy against cancer: mechanisms of resistance to oncolytic viruses. Future Oncol (2022) 18:245–59. doi: 10.2217/fon-2021-0802 34821517

[169] Fukuhara H Ino Y Todo T. Oncolytic virus therapy: A new era of cancer treatment at dawn. Cancer Sci (2016) 107:1373–9. doi: 10.1111/cas.13027 PMC5084676 27486853

[170] Lawler SE Speranza M-C Cho C-F Chiocca EA. Oncolytic viruses in cancer treatment: A review. JAMA Oncol (2017) 3:841–9. doi: 10.1001/jamaoncol.2016.2064 27441411

[171] Mardi A Shirokova AV Mohammed RN Keshavarz A Zekiy AO Thangavelu L. Biological causes of immunogenic cancer cell death (ICD) and anti-tumor therapy; combination of oncolytic virus-based immunotherapy and CAR T-cell therapy for ICD induction. Cancer Cell Int (2022) 22:168. doi: 10.1186/s12935-022-02585-z 35488303 PMC9052538

[172] Wolters JEJ van Mechelen RJS Al Majidi R Pinchuk L Webers CAB Beckers HJM. History, presence, and future of mitomycin c in glaucoma filtration surgery. Curr Opin Ophthalmol (2021) 32:148–59. doi: 10.1097/ICU.0000000000000729 33315724

[173] Bayat Mokhtari R Homayouni TS Baluch N Morgatskaya E Kumar S Das B. Combination therapy in combating cancer. Oncotarget (2017) 8:38022–43. doi: 10.18632/oncotarget.16723 PMC5514969 28410237

[174] Palmer AC Sorger PK. Combination cancer therapy can confer benefit *via* patient-to-Patient variability without drug additivity or synergy. Cell (2017) 171:1678–1691.e13. doi: 10.1016/j.cell.2017.11.009 29245013 PMC5741091

[175] Galoczova M Coates P Vojtesek B. STAT3, stem cells, cancer stem cells and p63. Cell Mol Biol Lett (2018) 23:12. doi: 10.1186/s11658-018-0078-0 29588647 PMC5863838

[176] Lee H Jeong AJ Ye SK. Highlighted STAT3 as a potential drug target for cancer therapy. BMB Rep (2019) 52(7):415–23. doi: 10.5483/BMBRep.2019.52.7.152 PMC6675244 31186087

[177] Adachi M Cui C Dodge CT Bhayani MK Lai SY. Targeting STAT3 inhibits growth and enhances radiosensitivity in head and neck squamous cell carcinoma. Oral Oncol (2012) 48:1220–6. doi: 10.1016/j.oraloncology.2012.06.006 PMC3734563 22770899

[178] Shao X Wang X Guo X Jiang K Ye T Chen J. STAT3 contributes to oncolytic Newcastle disease virus-induced immunogenic cell death in melanoma cells. Front Oncol (2019) 9:436. doi: 10.3389/fonc.2019.00436 31192135 PMC6548873

[179] Jafari S Lavasanifar A Hejazi MS Maleki-Dizaji N Mesgari M Molavi O. STAT3 inhibitory stattic enhances immunogenic cell death induced by chemotherapy in cancer cells. Daru (2020) 28:159–69. doi: 10.1007/s40199-020-00326-z PMC7214602 31942696

[180] Solari JIG Filippi-Chiela E Pilar ES Nunes V Gonzalez EA Figueiró F. Damage-associated molecular patterns (DAMPs) related to immunogenic cell death are differentially triggered by clinically relevant chemotherapeutics in lung adenocarcinoma cells. BMC Cancer (2020) 20:474. doi: 10.1186/s12885-020-06964-5 32456685 PMC7251700

[181] Baskar R Lee KA Yeo R Yeoh K-W. Cancer and radiation therapy: current advances and future directions. Int J Med Sci (2012) 9:193–9. doi: 10.7150/ijms.3635 PMC3298009 22408567

[182] Petersen SH Kua LF Nakajima S Yong WP Kono K. Chemoradiation induces upregulation of immunogenic cell death-related molecules together with increased expression of PD-L1 and galectin-9 in gastric cancer. Sci Rep (2021) 11:12264. doi: 10.1038/s41598-021-91603-7 34112882 PMC8192931

[183] Bains SJ Abrahamsson H Flatmark K Dueland S Hole KH Seierstad T. Immunogenic cell death by neoadjuvant oxaliplatin and radiation protects against metastatic failure in high-risk rectal cancer. Cancer Immunol Immunother (2020) 69:355–64. doi: 10.1007/s00262-019-02458-x PMC7044156 31893287

[184] Jin C Wang Y Li Y Li J Zhou S Yu J. Doxorubicin-near infrared dye conjugate induces immunogenic cell death to enhance cancer immunotherapy. Int J Pharm (2021) 607:121027. doi: 10.1016/j.ijpharm.2021.121027 34418473

[185] Limagne E Thibaudin M Nuttin L Spill A Derangère V Fumet J-D. Trifluridine/Tipiracil plus oxaliplatin improves PD-1 blockade in colorectal cancer by inducing immunogenic cell death and depleting macrophages. Cancer Immunol Res (2019) 7:1958–69. doi: 10.1158/2326-6066.CIR-19-0228 31611243

[186] Fukushima H Yoshida S Kijima T Nakamura Y Fukuda S Uehara S. Combination of cisplatin and irradiation induces immunogenic cell death and potentiates postirradiation anti-PD-1 treatment efficacy in urothelial carcinoma. Int J Mol Sci (2021) 22:e535. doi: 10.3390/ijms22020535 PMC7825793 33430352

[187] Zheng J Sun J Chen J Zhu S Chen S Liu Y. Oxygen and oxaliplatin-loaded nanoparticles combined with photo-sonodynamic inducing enhanced immunogenic cell death in syngeneic mouse models of ovarian cancer. J Control Release (2021) 332:448–59. doi: 10.1016/j.jconrel.2021.02.032 33662456

[188] Werthmöller N Frey B Rückert M Lotter M Fietkau R Gaipl US. Combination of ionising radiation with hyperthermia increases the immunogenic potential of B16-F10 melanoma cells *in vitro* and *in vivo* . Int J Hyperthermia (2016) 32:23–30. doi: 10.3109/02656736.2015.1106011 26754406

[189] Qi J Jin F Xu X Du Y. Combination cancer immunotherapy of nanoparticle-based immunogenic cell death inducers and immune checkpoint inhibitors. Int J Nanomed (2021) 16:1435–56. doi: 10.2147/IJN.S285999 PMC7910111 33654395

[190] Tatsuno K Yamazaki T Hanlon D Han P Robinson E Sobolev O. Extracorporeal photochemotherapy induces bona fide immunogenic cell death. Cell Death Dis (2019) 10:578. doi: 10.1038/s41419-019-1819-3 31371700 PMC6675789

[191] Turubanova VD Mishchenko TA Balalaeva IV Efimova I Peskova NN Klapshina LG. Novel porphyrazine-based photodynamic anti-cancer therapy induces immunogenic cell death. Sci Rep (2021) 11:7205. doi: 10.1038/s41598-021-86354-4 33785775 PMC8010109

[192] Ogawa M Tomita Y Nakamura Y Lee M-J Lee S Tomita S. Immunogenic cancer cell death selectively induced by near infrared photoimmunotherapy initiates host tumor immunity. Oncotarget (2017) 8:10425–36. doi: 10.18632/oncotarget.14425 PMC5354669 28060726

[193] Katsube R Noma K Ohara T Nishiwaki N Kobayashi T Komoto S. Fibroblast activation protein targeted near infrared photoimmunotherapy (NIR PIT) overcomes therapeutic resistance in human esophageal cancer. Sci Rep (2021) 11:1693. doi: 10.1038/s41598-021-81465-4 33462372 PMC7814141

[194] Guo S Jing Y Burcus NI Lassiter BP Tanaz R Heller R. Nano-pulse stimulation induces potent immune responses, eradicating local breast cancer while reducing distant metastases. Int J Cancer (2018) 142:629–40. doi: 10.1002/ijc.31071 28944452

[195] Guan J Wu Y Liu X Wang H Ye N Li Z. A novel prodrug and its nanoformulation suppress cancer stem cells by inducing immunogenic cell death and inhibiting indoleamine 2, 3-dioxygenase. Biomaterials (2021) 279:121180. doi: 10.1016/j.biomaterials.2021.121180 34768152

[196] Zhou M Luo C Zhou Z Li L Huang Y. Improving anti-PD-L1 therapy in triple negative breast cancer by polymer-enhanced immunogenic cell death and CXCR4 blockade. J Control Release (2021) 334:248–62. doi: 10.1016/j.jconrel.2021.04.029 33915224

[197] Guo J Yu Z Sun D Zou Y Liu Y Huang L. Two nanoformulations induce reactive oxygen species and immunogenetic cell death for synergistic chemo-immunotherapy eradicating colorectal cancer and hepatocellular carcinoma. Mol Cancer (2021) 20:10. doi: 10.1186/s12943-020-01297-0 33407548 PMC7786897

[198] Xu C Ju D Zhang X. Cell membrane-derived vesicle: A novel vehicle for cancer immunotherapy. Front Immunol (2022) 13:923598. doi: 10.3389/fimmu.2022.923598 35874757 PMC9300949

[199] Wan J Wang J Zhou M Rao Z Ling X. A cell membrane vehicle co-delivering sorafenib and doxorubicin remodel the tumor microenvironment and enhance immunotherapy by inducing immunogenic cell death in lung cancer cells. J Mater Chem B (2020) 8:7755–65. doi: 10.1039/d0tb01052a 32735004

[200] Zhou W Zhou Y Chen X Ning T Chen H Guo Q. Pancreatic cancer-targeting exosomes for enhancing immunotherapy and reprogramming tumor microenvironment. Biomaterials (2021) 268:120546. doi: 10.1016/j.biomaterials.2020.120546 33253966

[201] Liang Y Duan L Lu J Xia J. Engineering exosomes for targeted drug delivery. Theranostics (2021) 11:3183–95. doi: 10.7150/thno.52570 PMC7847680 33537081

[202] Zhu C Guo X Luo L Wu Z Luo Z Jiang M. Extremely effective chemoradiotherapy by inducing immunogenic cell death and radio-triggered drug release under hypoxia alleviation. ACS Appl Mater Interfaces (2019) 11:46536–47. doi: 10.1021/acsami.9b16837 31751119

[203] Alzeibak R Mishchenko TA Shilyagina NY Balalaeva IV Vedunova MV Krysko DV. Targeting immunogenic cancer cell death by photodynamic therapy: past, present and future. J Immunother Cancer (2021) 9:e001926. doi: 10.1136/jitc-2020-001926 33431631 PMC7802670

[204] Chhatre S Murgu S Vachani A Jayadevappa R. Photodynamic therapy for stage I and II non-small cell lung cancer: A SEER-Medicare analysis 2000-2016. Med (Baltimore) (2022) 101:e29053. doi: 10.1097/MD.0000000000029053 PMC10684201 35356921

[205] Candela L Kasraeian A Barret E. Current evidence for focal laser ablation and vascular-targeted photodynamic therapy for localized prostate cancer: review of literature published in the last 2 years. Curr Opin Urol (2022) 32:192–8. doi: 10.1097/MOU.0000000000000964 35013079

[206] Zhan Q Wu C Ding H Huang Y Jiang Z Liao N. Emerging trends in photodynamic therapy for head and neck cancer: A 10-year bibliometric analysis based on CiteSpace. Photodiagnosis Photodyn Ther (2022) 38:102860. doi: 10.1016/j.pdpdt.2022.102860 35429646

[207] Wang X-Y Maswikiti EP Zhu J-Y Ma Y-L Zheng P Yu Y. Photodynamic therapy combined with immunotherapy for an advanced esophageal cancer with an obstruction post metal stent implantation: A case report and literature review. Photodiagnosis Photodyn Ther (2022) 37:102671. doi: 10.1016/j.pdpdt.2021.102671 34864195

[208] Paraboschi I Turnock S Kramer-Marek G Musleh L Barisa M Anderson J. Near-InfraRed PhotoImmunoTherapy (NIR-PIT) for the local control of solid cancers: Challenges and potentials for human applications. Crit Rev Oncol Hematol (2021) 161:103325. doi: 10.1016/j.critrevonc.2021.103325 33836238 PMC8177002

[209] Furusawa A Okada R Inagaki F Wakiyama H Kato T Furumoto H. CD29 targeted near-infrared photoimmunotherapy (NIR-PIT) in the treatment of a pigmented melanoma model. Oncoimmunology (2022) 11:2019922. doi: 10.1080/2162402X.2021.2019922 35003897 PMC8741294

[210] Fukushima H Turkbey B Pinto PA Furusawa A Choyke PL Kobayashi H. Near-infrared photoimmunotherapy (NIR-PIT) in urologic cancers. Cancers (Basel) (2022) 14:2996. doi: 10.3390/cancers14122996 35740662 PMC9221010

[211] Nishimura T Mitsunaga M Ito K Kobayashi H Saruta M. Cancer neovasculature-targeted near-infrared photoimmunotherapy (NIR-PIT) for gastric cancer: different mechanisms of phototoxicity compared to cell membrane-targeted NIR-PIT. Gastric Cancer (2020) 23:82–94. doi: 10.1007/s10120-019-00988-y 31302791 PMC8189161

[212] Okada R Furusawa A Inagaki F Wakiyama H Kato T Okuyama S. Endoscopic near-infrared photoimmunotherapy in an orthotopic head and neck cancer model. Cancer Sci (2021) 112:3041–9. doi: 10.1111/cas.15013 PMC8353912 34101947

[213] Kobayashi H Furusawa A Rosenberg A Choyke PL. Near-infrared photoimmunotherapy of cancer: a new approach that kills cancer cells and enhances anti-cancer host immunity. Int Immunol (2020) 33:7–15. doi: 10.1093/intimm/dxaa037 PMC7771006 32496557

[214] Cognetti DM Johnson JM Curry JM Kochuparambil ST McDonald D Mott F. Phase 1/2a, open-label, multicenter study of RM-1929 photoimmunotherapy in patients with locoregional, recurrent head and neck squamous cell carcinoma. Head Neck (2021) 43:3875–87. doi: 10.1002/hed.26885 PMC9293150 34626024

[215] Ye Y Zhao Y Sun Y Cao J. Recent progress of metal-organic framework-based photodynamic therapy for cancer treatment. Int J Nanomed (2022) 17:2367–95. doi: 10.2147/IJN.S362759 PMC9144878 35637838

[216] Nuccitelli R McDaniel A Connolly R Zelickson B Hartman H. Nano-pulse stimulation induces changes in the intracellular organelles in rat liver tumors treated in situ. Lasers Surg Med (2020) 52:882–9. doi: 10.1002/lsm.23239 PMC7586959 32220023

[217] Nuccitelli R. Application of pulsed electric fields to cancer therapy. Bioelectricity (2019) 1:30–4. doi: 10.1089/bioe.2018.0001 PMC8370244 34471806

[218] Beebe SJ Lassiter BP Guo S. Nanopulse stimulation (NPS) induces tumor ablation and immunity in orthotopic 4T1 mouse breast cancer: A review. Cancers (Basel) (2018) 10:e97. doi: 10.3390/cancers10040097 PMC5923352 29601471

[219] Nguyen HTM Katta N Widman JA Takematsu E Feng X Torres-Hurtado SA. Laser nanobubbles induce immunogenic cell death in breast cancer. Nanoscale (2021) 13:3644–53. doi: 10.1039/d0nr06587k PMC8710258 33538275

[220] Tian M Xin X Wu R Guan W Zhou W. Advances in intelligent-responsive nanocarriers for cancer therapy. Pharmacol Res (2022) 178:106184. doi: 10.1016/j.phrs.2022.106184 35301111

[221] Vincent MP Navidzadeh JO Bobbala S Scott EA. Leveraging self-assembled nanobiomaterials for improved cancer immunotherapy. Cancer Cell (2022) 40:255–76. doi: 10.1016/j.ccell.2022.01.006 PMC8930620 35148814

[222] Yan W Lang T Qi X Li Y. Engineering immunogenic cell death with nanosized drug delivery systems improving cancer immunotherapy. Curr Opin Biotechnol (2020) 66:36–43. doi: 10.1016/j.copbio.2020.06.007 32673944

[223] Appiah E Nakamura H Pola R Grossmanová E Lidický O Kuniyasu A. Acid-responsive HPMA copolymer-bradykinin conjugate enhances tumor-targeted delivery of nanomedicine. J Control Release (2021) 337:546–56. doi: 10.1016/j.jconrel.2021.08.009 34375687

[224] Rani S Gupta U. HPMA-based polymeric conjugates in anticancer therapeutics. Drug Discovery Today (2020) 25:997–1012. doi: 10.1016/j.drudis.2020.04.007 32334073

[225] De Jong WH Borm PJA. Drug delivery and nanoparticles:applications and hazards. Int J Nanomed (2008) 3:133–49. doi: 10.2147/ijn.s596 PMC2527668 18686775

[226] Feng S Ren Y Li H Tang Y Yan J Shen Z. Cancer cell-membrane biomimetic boron nitride nanospheres for targeted cancer therapy. Int J Nanomed (2021) 16:2123–36. doi: 10.2147/IJN.S266948 PMC7959002 33731994

[227] Chen H-Y Deng J Wang Y Wu C-Q Li X Dai H-W. Hybrid cell membrane-coated nanoparticles: A multifunctional biomimetic platform for cancer diagnosis and therapy. Acta Biomater (2020) 112:1–13. doi: 10.1016/j.actbio.2020.05.028 32470527

[228] Pegtel DM Gould SJ. Exosomes. Annu Rev Biochem (2019) 88:487–514. doi: 10.1146/annurev-biochem-013118-111902 31220978

[229] Kalluri R LeBleu VS. The biology, function, and biomedical applications of exosomes. Science (2020) 367:eaau6977. doi: 10.1126/science.aau6977 32029601 PMC7717626

[230] Gorain B Al-Dhubiab BE Nair A Kesharwani P Pandey M Choudhury H. Multivesicular liposome: A lipid-based drug delivery system for efficient drug delivery. Curr Pharm Des (2021) 27:4404–15. doi: 10.2174/1381612827666210830095941 34459377

[231] Almeida B Nag OK Rogers KE Delehanty JB. Recent progress in bioconjugation strategies for liposome-mediated drug delivery. Molecules (2020) 25:e5672. doi: 10.3390/molecules25235672 PMC7730700 33271886

[232] Liu D Chen B Mo Y Wang Z Qi T Zhang Q. Redox-activated porphyrin-based liposome remote-loaded with indoleamine 2,3-dioxygenase (IDO) inhibitor for synergistic photoimmunotherapy through induction of immunogenic cell death and blockage of IDO pathway. Nano Lett (2019) 19:6964–76. doi: 10.1021/acs.nanolett.9b02306 31518149

[233] Hoshyar N Gray S Han H Bao G. The effect of nanoparticle size on *in vivo* pharmacokinetics and cellular interaction. Nanomed (Lond) (2016) 11:673–92. doi: 10.2217/nnm.16.5 PMC5561790 27003448

[234] Dobrovolskaia MA Aggarwal P Hall JB McNeil SE. Preclinical studies to understand nanoparticle interaction with the immune system and its potential effects on nanoparticle biodistribution. Mol Pharm (2008) 5:487–95. doi: 10.1021/mp800032f PMC2613572 18510338

[235] Labouta HI Asgarian N Rinker K Cramb DT. Meta-analysis of nanoparticle cytotoxicity *via* data-mining the literature. ACS Nano (2019) 13:1583–94. doi: 10.1021/acsnano.8b07562 30689359

[236] Antoniotti C Borelli B Rossini D Pietrantonio F Morano F Salvatore L. AtezoTRIBE: a randomised phase II study of FOLFOXIRI plus bevacizumab alone or in combination with atezolizumab as initial therapy for patients with unresectable metastatic colorectal cancer. BMC Cancer (2020) 20:683. doi: 10.1186/s12885-020-07169-6 32698790 PMC7376656

[237] Bernal-Estévez DA Ortíz Barbosa MA Ortíz-Montero P Cifuentes C Sánchez R Parra-López CA. Autologous dendritic cells in combination with chemotherapy restore responsiveness of T cells in breast cancer patients: A single-arm phase I/II trial. Front Immunol (2021) 12:669965. doi: 10.3389/fimmu.2021.669965 34489928 PMC8417880

[238] Cibula D Rob L Mallmann P Knapp P Klat J Chovanec J. Dendritic cell-based immunotherapy (DCVAC/OvCa) combined with second-line chemotherapy in platinum-sensitive ovarian cancer (SOV02): A randomized, open-label, phase 2 trial. Gynecol Oncol (2021) 162:652–60. doi: 10.1016/j.ygyno.2021.07.003 34294416

[239] Lin M Zhang X Liang S Luo H Alnaggar M Liu A. Irreversible electroporation plus allogenic Vγ9Vδ2 T cells enhances antitumor effect for locally advanced pancreatic cancer patients. Signal Transduct Target Ther (2020) 5:215. doi: 10.1038/s41392-020-00260-1 33093457 PMC7582168

[240] Lang FF Conrad C Gomez-Manzano C Yung WKA Sawaya R Weinberg JS. Phase I study of DNX-2401 (Delta-24-RGD) oncolytic adenovirus: Replication and immunotherapeutic effects in recurrent malignant glioma. J Clin Oncol (2018) 36:1419–27. doi: 10.1200/JCO.2017.75.8219 PMC6075856 29432077

[241] Packiriswamy N Upreti D Zhou Y Khan R Miller A Diaz RM. Oncolytic measles virus therapy enhances tumor antigen-specific T-cell responses in patients with multiple myeloma. Leukemia (2020) 34:3310–22. doi: 10.1038/s41375-020-0828-7 PMC7581629 32327728

